# Virtual Reality and Transcranial Direct Current Stimulation for Posttraumatic Stress Disorder: A Randomized Clinical Trial

**DOI:** 10.1001/jamapsychiatry.2023.5661

**Published:** 2024-03-06

**Authors:** Mascha van ’t Wout-Frank, Amanda R. Arulpragasam, Christiana Faucher, Emily Aiken, M. Tracie Shea, Richard N. Jones, Benjamin D. Greenberg, Noah S. Philip

**Affiliations:** 1 Center for Neurorestoration and Neurotechnology, VA Providence Healthcare System, Providence, Rhode Island; 2 Department of Psychiatry and Human Behavior, Alpert Medical School of Brown University, Providence, Rhode Island

## Abstract

**Importance:**

Posttraumatic stress disorder (PTSD) is a common psychiatric disorder that is particularly difficult to treat in military veterans. Noninvasive brain stimulation has significant potential as a novel treatment to reduce PTSD symptoms.

**Objective:**

To test whether active transcranial direct current stimulation (tDCS) plus virtual reality (VR) is superior to sham tDCS plus VR for warzone-related PTSD.

**Design, Setting, and Participants:**

This double-blind randomized clinical trial was conducted among US military veterans enrolled from April 2018 to May 2023 at a secondary care Department of Veterans Affairs hospital and included 1- and 3-month follow-up visits. Participants included US military veterans with chronic PTSD and warzone-related exposure, recruited via referral and advertisement. Patients in psychiatric treatment had to be on a stable regimen for at least 6 weeks to be eligible for enrollment. Data were analyzed from May to September 2023.

**Intervention:**

Participants were randomly assigned to receive 2-mA anodal tDCS or sham tDCS targeted to the ventromedial prefrontal cortex, during six 25-minute sessions of standardized warzone VR exposure, delivered over 2 to 3 weeks.

**Main Outcomes and Measures:**

The co–primary outcomes were self-reported PTSD symptoms, measured via the PTSD checklist for *DSM-5* (PCL-5), alongside quality of life. Other outcomes included psychophysiological arousal, clinician-assessed PTSD, depression, and social/occupational function.

**Results:**

A total of 54 participants (mean [SD] age, 45.7 [10.5] years; 51 [94%] males) were assessed, including 26 in the active tDCS group and 28 in the sham tDCS group. Participants in the active tDCS group reported a superior reduction in self-reported PTSD symptom severity at 1 month (*t* = −2.27, *P* = .02; Cohen *d* = −0.82). There were no significant differences in quality of life between active and sham tDCS groups. Active tDCS significantly accelerated psychophysiological habituation to VR events between sessions compared with sham tDCS (*F*_5,7689.8_ = 4.65; *P* < .001). Adverse effects were consistent with the known safety profile of the corresponding interventions.

**Conclusions and Relevance:**

These findings suggest that combined tDCS plus VR may be a promising strategy for PTSD reduction and underscore the innovative potential of these combined technologies.

**Trial Registration:**

ClinicalTrials.gov Identifier: NCT03372460

## Key Points

**Question:**

Can therapeutic exposure using virtual reality (VR) be augmented with simultaneously applied transcranial direct current stimulation (tDCS) to reduce symptoms of posttraumatic stress disorder (PTSD)?

**Findings:**

In this randomized clinical trial including 54 US military veterans with warzone-related trauma, active tDCS delivered during VR exposure significantly improved self-reported PTSD symptoms, reduced measures of autonomic arousal, and improved social functioning compared with sham stimulation during VR exposure. This trial replicated key findings from a prior pilot study.

**Meaning:**

These findings suggest that the use of combined VR exposure plus tDCS could be a promising treatment for warzone-related PTSD.

## Teaser

This randomized clinical trial assesses whether active transcranial direct current stimulation plus virtual reality is effective in treating warzone-related PTSD compared with sham stimulation plus virtual reality.

## Introduction

Posttraumatic stress disorder (PTSD), characterized by intrusive thoughts and recollections, avoidance of trauma-related stimuli, hyperarousal, and disturbed mood and cognitions, is highly prevalent and disabling.^1,2^ It is associated with comorbid medical and psychiatric disorders, substance use, and suicide.^3,4,5^ Rates of PTSD, and its associated burdens, are particularly high in veterans.^6,7^

First-line PTSD treatments include trauma-focused cognitive behavioral therapies and selective serotonin or serotonin-norepinephrine reuptake inhibitors.^8^ Unfortunately, many individuals do not meaningfully benefit from these treatments. Nonresponse to gold-standard exposure therapy is substantial, and up to 50% of patients drop out, perhaps reflecting inability to tolerate negative effects during exposures.^9,10,11^ Medications have moderate efficacy for PTSD^12^ and have significant adverse effects. Furthermore, treatment effectiveness is reduced in veterans.^12^

A leading theory of PTSD pathophysiology posits that fear extinction and extinction retention are impaired due to ineffective top-down control of the amygdala by ventromedial prefrontal cortex (VMPFC) and other regions.^13,14^ Affected individuals thus have impaired safety learning and memory, which in healthy people is supported by intact VMPFC-amygdala circuit function.^15,16,17^ Thus, enhancing inhibitory VMPFC modulation of the amygdala^18^ might improve safety learning and reconsolidation during exposure and thereby improve PTSD symptoms.

Noninvasive transcranial direct current stimulation (tDCS) is well suited to potentially augment trauma-focused exposure therapy. In tDCS, weak electric currents applied at the scalp are thought to bias neuronal firing to ongoing inputs.^19^ Anodal tDCS, which can be thought to facilitate ongoing neural activity, might augment learning and retention of safety memories, accelerating fear extinction.^20^ tDCS targeting VMPFC may improve extinction learning in healthy individuals.^21,22,23^ tDCS applied following extinction learning (ie, during synaptic consolidation) may boost extinction memory in veterans with PTSD.^24^

These studies used neutral stimuli in a Pavlovian extinction paradigm, which does not capture emotional intensity or context-specific trauma memory cues. One novel tool to manipulate contextual presentation in mental health research and treatment is virtual reality (VR), which provides an immersive experience to simulated environments or stimuli, often in a graded fashion, that are otherwise inaccessible or cannot be replicated during exposure.^25^ With this in mind, we previously conducted a pilot single-blind trial of sham-controlled tDCS plus virtual reality (VR) exposure to warzone cues in veterans with PTSD,^26^ inclusive of 6 VR sessions over 2 weeks. Active tDCS plus VR, compared with sham tDCS plus VR, resulted in a greater decrease in psychophysiological arousal across the 6 treatment sessions, indicating between-session habituation. All participants had meaningful reductions in PTSD symptoms, attributed to the VR procedure. These findings prompted this better-powered, double-blind, randomized clinical trial testing the hypothesis that active tDCS would augment the effects of VR to improve PTSD severity, physiological arousal, and function.

## Methods

For this randomized clinical trial, all procedures were approved by the VA Providence Healthcare System institutional review board. All participants provided written informed consent. The trial protocol and statistical analysis plan are provided in Supplement 1. This study is reported following the Consolidated Standards of Reporting Trials (CONSORT) reporting guideline.

**Figure 1.**
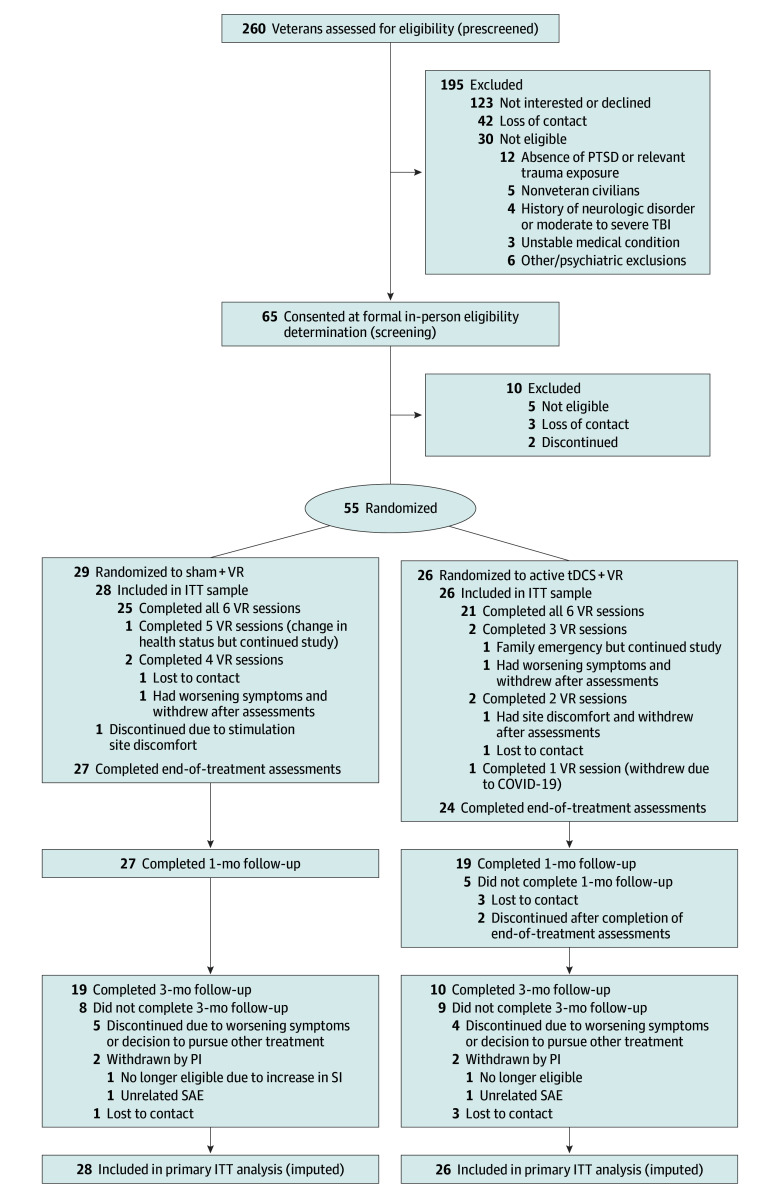
Participant Recruitment and Randomization Flowchart ITT indicates intention to treat; PI, principal investigator; PTSD, posttraumatic stress disorder; SAE, serious adverse event; TBI, traumatic brain injury; tDCS, transcranial direct current stimulation; VR, virtual reality.

Participants were recruited from the VA Providence Healthcare System from April 2018 to May 2023. Recruitment ended at funding completion. Among 65 participants who provided written informed consent, 54 were evaluated in a modified intent-to-treat fashion (ie, provided informed consent, were randomized, and completed ≥1 VR session) (Figure 1). The principal inclusion criteria were a diagnosis of chronic PTSD by *DSM-5* criteria (assessed via the Clinician-Administered PTSD Scale for *DSM-5* [CAPS-5]^27^) with warzone-related trauma, with other diagnoses assessed via the Mini International Neuropsychiatric Interview.^28^ Warzone experience was intentionally chosen to include those with combat and noncombat experience, since many veterans do not experience direct combat yet are exposed to trauma in a warzone environment or while deployed. Other trauma (eg, prior to the military) was not exclusory. Eligible participants of any sex or gender were between ages 18 to 65 years. If a patient was in psychiatric treatment, they had to be maintaining a stable regimen for more than 6 weeks (and stable until at least the 1-month time point) for eligibility. Exclusion criteria were neuroimaging or tDCS contraindications; pregnancy, lactation, or planning to become pregnant; bipolar I disorder; moderate or severe traumatic brain injury; medically unstable or significant neurological disorders; primary psychotic disorders; active moderate or severe substance use disorders (within the last month, excluding nicotine and caffeine); or active suicidal intent or plan.

Participant demographic characteristics were obtained at baseline. Race and ethnicity were assessed and categorized by self-report, with race categorized as African American, Asian, multiracial, or White and ethnicity categorized as Hispanic or not Hispanic. Race and ethnicity were included in analysis because of the importance of consideration of race and ethnicity in research.

### Randomization

Eligible participants were randomized to active tDCS plus VR or sham tDCS plus VR using a 1:1 ratio stratified on sex (male or female) and self-reported PTSD symptom severity (assessed with PTSD Checklist for *DSM-5* [PCL-5],^29^ with score <48 indicating low and 49-80, high) based on Philip et al,^30^ to increase likelihood of balance across treatment groups. Randomization was blocked using random block sizes of 2 and 4. A study investigator not involved with enrollment generated the allocation sequence; participants and staff were blinded to allocation.

### Intervention

Using a parallel-group double-blind, sham-controlled design, we delivered combined active or sham tDCS plus VR for up to 6 sessions over 10 business days, with at least 1 day between sessions. Each session lasted approximately 60 minutes, inclusive of study setup, active or sham tDCS plus VR, and postsession debriefing. An overview of the methods is provided in the eFigure in Supplement 2.

#### Transcranial Direct Current Stimulation Procedures

tDCS was delivered using a NeuroConn DC-Stimulator Plus (NeuroConn). Electrical field modeling (tDCS/HD Explore version 4.0; Soterix Medical) indicated that anode placement over 10 to 20 electroencephalography (EEG) coordinate AF7, Fp1, and AF3 and cathode between EEG coordinate Oz and the contralateral mastoid (covering approximately PO8 and P8) resulted in the highest electrical field values in the VMPFC region associated with extinction extracted from peak voxels in an imaging meta-analysis.^31^ We placed 3 × 3-cm electrodes (current density of 2.22 A/m^2^) in a reusable sponge saturated with 0.9% normal saline and held in place with a rubber headband. After setup, staff assessed tolerability by applying a brief stimulation (1 mA for 30 seconds, with 30-second ramp up and ramp down). Stimulation started simultaneously with VR and continued throughout the session. Active tDCS was 2mA for 25 minutes with a 30-second ramp up and ramp down, and sham stimulation was provided through the NeuroConn study mode (10 μA over 15 milliseconds current pulse applied every 550 milliseconds, 3 ms peak current).^32^

#### Virtual Reality Procedures

We used the Bravemind VR application version 1.0.4 or higher (Virtually Better) for VR. This system was designed with input from veterans with lived experience, and provides a visual, auditory, haptic, and olfactory immersion into virtual Iraq or Afghanistan. Procedures followed the pilot study^26,32^ using a standardized driving scenario that most closely matched their deployment (ie, Iraq or Afghanistan), following Difede and Hoffman^33^ and by Rothbaum and colleagues.^34^

As in our pilot study, participants were presented with 12 VR events that started with a low-intensity VR experience of riding in a mine-resistant ambush-protected (MRAP) vehicle with escalating exposure, including smell of weapons fire paralleling VR exposure therapy (VR events were, in order: distant gun battle, A-10 flyover, 0.50-caliber burst, Black Hawk flyover, road ambush 1, road ambush 2, improvised explosive device [IED] at right with 40-m distance, road ambush, IED at right with 40-m distance, IED at left with 30-m distance, bridge ambush, IED in front with lead vehicle flip). Presentation of discrete VR events was timed with respect to previous stimulus elements to obtain stimulus-related psychophysiological responses. Participants repeated the same 8-minute VR scenario 3 times over approximately 25 minutes with approximately 30 seconds between scenarios to check how participants tolerated the experience. They were asked to treat the scenes as real and could stop at any time.

### Skin Conductance

Examination of changes in psychophysiological arousal was one of the aims of the study. To assess changes in psychophysiological arousal among the 6 sessions and within each session (measuring between-session and within-session habituation), we used a Biopac MP-150 system (version RRID:SCR_014279 and 2 disposable (EL507) electrodes on the thenar eminence of the nondominant hand to record skin conductance (SC). Adequacy of electrode attachment and acquisition was confirmed by observing fluctuation in SC in response to taking a deep breath. SC level was recorded for 2 minutes before each VR session to establish a physiologic baseline, and throughout each session; trials with SC level less than 2 microsiemens, indicating inadequate acquisition, were excluded. Biopac AcqKnowledge software version 4.3 was used to detect and process fluctuations in SC during VR, taking peak SC value during 10 seconds following each VR event minus the minimum SC value during 1 second prior to that event.^26,35^ We log transformed SC data to reduce skew.

### Measures and Participant Assessments

The co–primary outcomes were self-reported PTSD symptoms and quality of life. Other measures, including SC, represented additional outcomes. Self-reported PTSD symptom severity was assessed using the PCL-5 at baseline, midpoint (after VR session 3), end point (after the last VR session), and at 1 and 3 months. A change of 10 or more points was defined as clinically meaningful.^29^ The PCL-5 was used as a primary measure because of its use in the VA system to inform treatment decisions in measurement-based care. Based on our pilot study, we hypothesized active tDCS plus VR would result in a greater reduction on the PCL-5 from baseline to the end of treatment and at the 1-month follow-up compared with sham stimulation plus VR. Quality of life was measured via the Short-Form Quality of Life Enjoyment and Satisfaction Questionnaire.^36^ Additional outcomes included self-reported depressive symptoms using the Inventory of Depressive Symptoms, Self-Report,^37^ assessed on the same schedule as the PCL-5. Other outcome measures, measured at baseline, after the last tDCS plus VR session, and at 1 and 3 months included clinician-assessed PTSD symptom severity (CAPS-5),^27^ quality of life,^38^ and clinician-assessed social and occupational functioning (assessed with Social and Occupational Function Scale).^39^ Safety was assessed via spontaneous report and a tDCS side effect checklist based on Brunoni et al.^40^ Blinding was assessed after completion of tDCS plus VR (eAppendix 1 in Supplement 2).

### Statistical Analysis

Clinical outcomes were analyzed with linear mixed-effect models to control for nonindependence of repeated observations with Stata software version 18 (StataCorp). Baseline value of the outcome and the interaction of a dummy variable for the active tDCS condition and dummy variables for time (with t1 indicating midpoint; t2, end point; t3, one-month follow-up; t4, three-month follow-up) were included as predictors. Missing data were addressed using multiple imputations (20 imputations). Skin conductance responses were separately analyzed using a linear mixed model with SPSS Statistics version 27 (IBM), including dummy variables tDCS group, VR session (1-6), run within session (1-3), and individual VR events (1-12) as predictors and SC response as independent variable.^41^ Participant was entered as a random-effects variable to control for nonindependence of repeated observations. Significance was set at a 2-tailed *P* < .05, and effect sizes were defined using Cohen *d*.^42^ Estimated sample size was informed by our prior research in this patient population.^41,43^ Data were analyzed from May to September 2023.

## Results

A total of 54 participants (mean [SD] age, 45.7 [10.5] years; 51 [94%] males) were assessed, including 26 in the active tDCS group and 28 in the sham tDCS group. Randomization resulted in groups balanced on demographic variables and PTSD symptom severity that did not meaningfully differ (Table 1; eTable 1 and eAppendix 2 in Supplement 2). Baseline PTSD scores were moderate, approximately half of participants had comorbid depression, and approximately half of participants had a prior mild traumatic brain injury (Table 1). Overall, 21 participants (39%) had a prior suicide attempt, and more than half of participants had a prior inpatient psychiatric hospitalization (Table 1). Baseline assessments also indicated low social and occupational function and poor quality of life. While warzone-related trauma was required for inclusion, trauma exposure was multifactorial across groups (eTable 2 in Supplement 2). Medication use did not differ by group: most participants were using multiple medications for PTSD and related comorbidities (Table 1).

**Table 1.** Participant Demographic and Clinical Features

Variable	Patients, No. (%)^a^	
	Active tDCS (n = 26)	Sham tDCS (n = 28)
Age, mean (SD), y	44 (12)	47 (9)
Sex		
Female	<3 (NR)	<3 (NR)
Male	25 (96)	26 (93)
Race		
African American	<3 (NR)^b^	<3 (NR)^b^
Asian	0	<3 (NR)^b^
Multiracial	<3 (NR)^b^	<3 (NR)^b^
White	21 (81)	22 (79)
Ethnicity		
Hispanic	<3 (NR)^b^	3 (11)
Not Hispanic	21 (81)	25 (89)
Education		
High school or equivalent	0	3 (11)
Some college	9 (35)	13 (46)
Trade or vocational degree	5 (19)	<3 (NR)^b^
Bachelor’s degree	5 (19)	5 (18)
Advanced degree and/or education beyond college	7 (27)	6 (21)
Employment status		
Full time	4 (15)	8 (29)
Part time	3 (12)	5 (18)
Unemployed	8 (31)	6 (21)
Retired	8 (31)	6 (21)
Disabled	14 (54)	12 (43)
Student	0	<3 (NR)^b^
Service-connected disability (mental health)	22 (85)	22 (79)
Military branch		
Army	16 (62)	17 (61)
Navy	<3 (NR)^b^	3 (11)
Marines	4 (15)	4 (14)
National Guard	<3 (NR)^b^	<3 (NR)^b^
Air Force	<3 (NR)	<3 (NR)^b^
Coast Guard	0	<3 (NR)^b^
Psychiatric comorbidities		
MDD	13 (50)	9 (32)
Alcohol use disorder	5 (19)	4 (14)
Substance use disorder (other)	5 (19)	<3 (NR)^b^
Panic disorder	9 (35)	4 (14)
Generalized anxiety disorder	5 (19)	8 (29)
Mild traumatic brain injury	10 (38)	16 (55)
Psychiatric history		
Suicide attempt	11 (42)	10 (34)
Inpatient hospitalization	15 (58)	16 (55)

Abbreviations: CAPS-5, Clinician-Administered PTSD Scale for *DSM-5*; IDSSR, Inventory of Depressive Symptomatology, Self-Report; MDD, major depressive disorder; NR, not reported; QLESQ, Quality of Life Enjoyment and Satisfaction Questionnaire; PCL-5, PTSD Checklist for *DSM-5*; PTSD, posttraumatic stress disorder; SOFAS, Social and Occupational Function Scale; tDCS, transcranial direct current stimulation.

^a^ Totals may not equal 100% due to nonresponse or multiple responses.

^b^ Not reported because there were too few patients to provide numbers without compromising identifiability.

### Clinical Outcomes

Participants were unable to accurately guess their assignment to active or sham tDCS (χ^2^ = 0.07; *P* = .96). Statistically significant reductions in PTSD symptoms from baseline emerged over time, favoring active tDCS plus VR (Table 2). Participants who received active tDCS plus VR reported meaningful PCL-5 severity improvement (>10-point reduction from baseline after 3 sessions, ie, the midpoint, and at the end of tDCS plus VR) compared with participants in the sham tDCS group. Participants who received active tDCS plus VR also had significantly greater reduction in PTSD symptom severity at the 1-month follow-up compared with the sham tDCS group, with a large effect size (*t* = −2.27; *P* = .023; Cohen *d* = −0.82). Although PTSD improvement continued over the 3-month follow-up, group differences were not statistically significant with a large effect size (*t* = −1.82; *P* = .07; Cohen *d* = −0.88). Insight into which participants reported changes in PTSD symptoms was gained via cluster analyses on PCL-5 improvements from baseline to 1-month follow-up (eAppendix 3 in Supplement 2).

**Table 2.** Clinical Outcomes Over Time Among Active tDCS Plus VR vs Sham tDCS Plus VR Participants

Outcome	Mean (SD) [No. of participants]		t Score	*P* value	Cohen *d*^a^
	Active tDCS + VR	Sham tDCS + VR			
**PCL-5** ^b^					
Baseline	48.6 (12.1) [26]	45.0 (11.9) [28]	NA	NA	NA
Midpoint	38.5 (14.4) [24]	41.3 (15.1) [28]	−1.34	.19	−0.46
End of VR sessions	36.0 (15.4) [24]	38.9 (15.1) [27]	−1.89	.06	−0.66
1 mo	31.4 (17.8) [19]	37.9 (16.6) [27]	−2.27	.02	−0.82
3 mo	21.2 (12.6) [10]	32.3 (17.1) [19]	−1.87	.07	−0.88
**CAPS-5** ^c^					
Baseline	44.8 (8.2) [26]	40.5 (8.1) [28]	NA	NA	NA
Midpoint	NA	NA	NA	NA	NA
End of VR sessions	40.4 (11.2) [24]	37.1 (9.2) [26]	−0.25	.80	−0.10
1 mo	34.1 (14.7) [19]	36.1 (12.6) [27]	−1.16	.25	−0.51
3 mo	22.7 (13.9) [9]	31 (11.9) [19]	−1.76	.08	−0.91
**QLESQ** ^c^					
Baseline	35.9 (8.9) [26]	39.2 (9.8) [28]	NA	NA	NA
Midpoint	NA	NA	NA	NA	NA
End of VR sessions	39.2 (10.2) [24]	41.6 (9.2) [26]	0.59	.55	0.14
1 mo	40.7 (10.3) [19]	41.0 (8.5) [27]	0.67	.50	0.16
3 mo	44.3 (9.6) [10]	47.1 (10.1) [19]	0.50	.61	0.19
**SOFAS** ^c^					
Baseline	40.5 (9.7) [26]	43.0 (11.2) [25]	NA	NA	NA
Midpoint	NA	NA	NA	NA	NA
End of VR sessions	47.2 (14.7) [23]	48.0 (12.2) [25]	0.34	.73	0.12
1 mo	53.1 (13.8) [28]	54.1 (15.4) [22]	1.32	.18	0.50
3 mo	67.2 (8.3) [9]	62.2 (15.3) [19]	2.78	.006	1.2
**IDSSR** ^b^					
Baseline	41.6 (12.0) [26]	38.6 (13.0) [28]	NA	NA	NA
Midpoint	35.5 (10.7) [23]	35.3 (13.4) [22]	−0.47	.64	−0.14
End of VR sessions	34.2 (15.1) [24]	34.8 (14.5) [26]	−0.64	.53	−0.19
1 mo	30.5 (16.2) [19]	32.3 (15.5) [27]	−0.71	.48	−0.23
3 mo	23.4 (12.7) [10]	27.1 (14.8) [19]	−0.99	.33	−0.35

Abbreviations: CAPS-5, Clinician-Administered PTSD Scale for *DSM-5*; IDSSR, Inventory of Depressive Symptomatology, Self-Report; NA, not applicable; PCL-5, PTSD Checklist for *DSM-5*; PTSD, posttraumatic stress disorder; QLESQ, Quality of Life Enjoyment and Satisfaction Questionnaire; SOFAS, Social and Occupational Function Scale; tDCS, transcranial direct current stimulation; VR, virtual reality.

^a^ Negative Cohen *d* represents a therapeutic reduction in scale value, whereas positive value of Cohen *d* represents increase in score (eg, reduction in PCL-5 score, and increase in SOFAS score, respectively).

^b^ PCL-5 and IDSSR were obtained at the end of every week.

^c^ CAPS-5, SOFAS, and QLESQ were obtained at the end of every 2 weeks.

**Figure 2.**
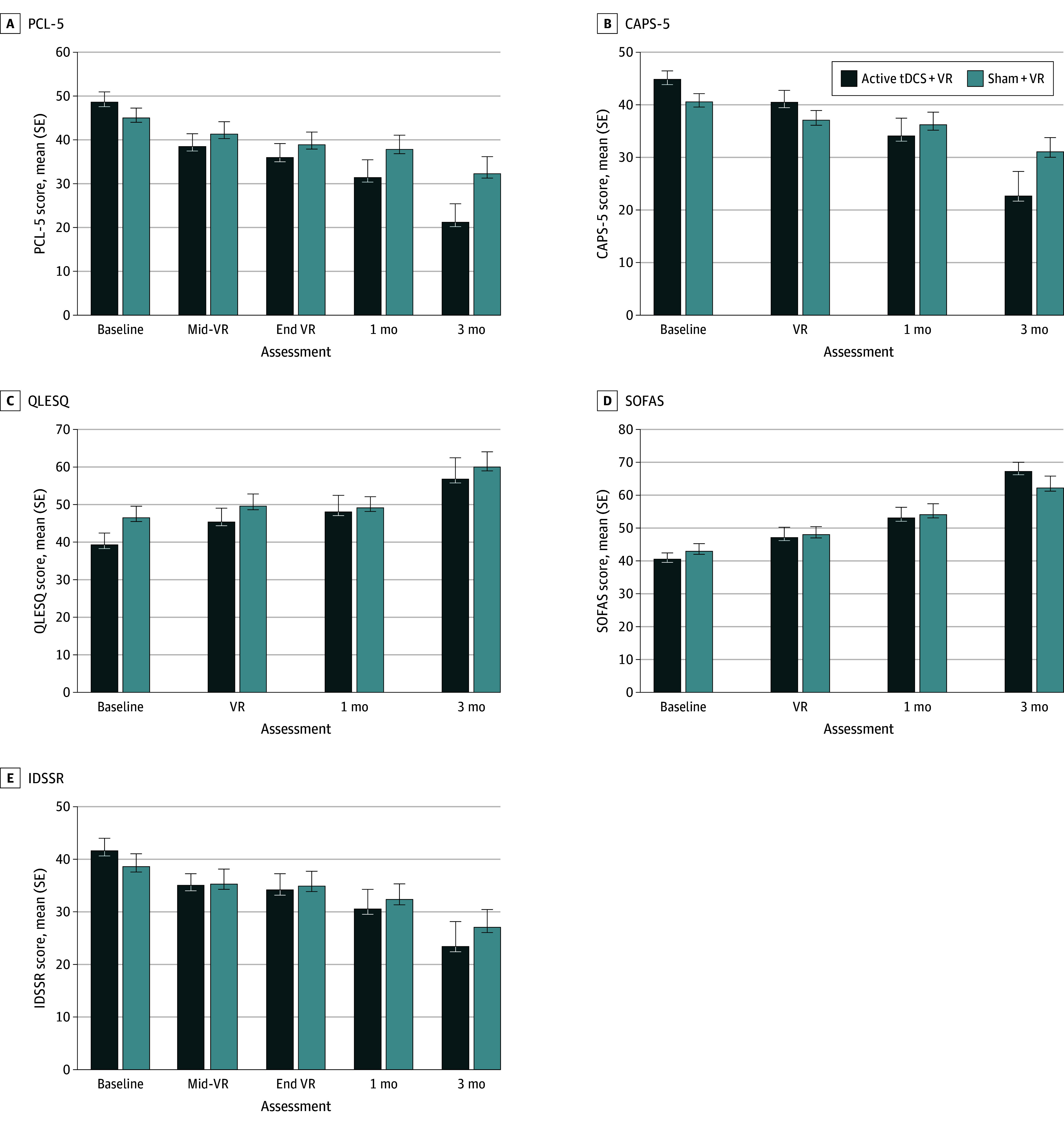
Clinical Outcomes Over Time Scores indicate nonimputed (raw) values over time. Error bars represent SE of the mean. CAPS-5 indicates Clinician-Administered Posttraumatic Stress Disorder (PTSD) Scale for *DSM-5*; IDSSR, Inventory of Depressive Symptomatology, Self-Report; PCL-5, PTSD Checklist for *DSM-5*; QLESQ, Quality of Life and Satisfaction Questionnaire (short form); tDCS, transcranial direct current stimulation; VR, virtual reality.

Symptom improvement in the active group was specific to self-reported PTSD; while depressive severity improved over time in both groups (*F*_4,49_ = 6.89; *P* < .001), no group differences were observed. Clinician-assessed PTSD via the CAPS-5 indicated no significant effect sizes regarding active tDCS at 1 and 3 months. Self-reported quality of life showed nonspecific improvement over time (*F*_3,50_ = 4.85; *P* = .005). Clinician-assessed social and occupational function showed significant and meaningful improvement at 3 months in the active tDCS group compared with the sham group (*t* = 2.78; *P* = .006; Cohen *d* = 1.2) (Figure 2).

### Psychophysiological Outcomes

**Figure 3.**
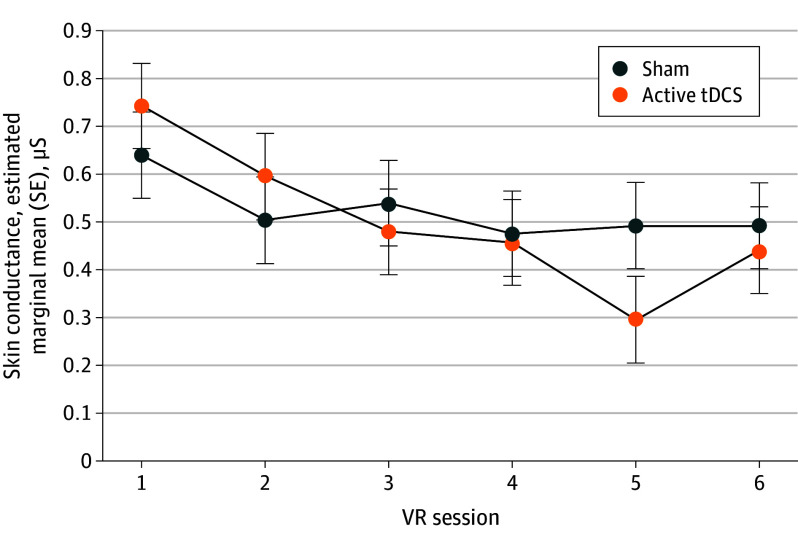
Changes in Skin Conductance Over Time Skin conductance reactivity (nontransformed in microsiemens [μS]) across VR sessions (1-6) for active transcranial direct current stimulation (tDCS) plus virtual reality (VR) and sham + VR groups. Error bars represent SE.

Reductions in SC reactivity across sessions were greater for the active tDCS plus VR group compared with the sham tDCS plus VR group, evidenced by a significant session by group interaction (*F*_5,7689.8_ = 4.65; *P* = <.001) (Figure 3), suggesting active tDCS augmented habituation across sessions. Results were robust to sensitivity analyses, including sessions with poor SC (eAppendix 4 in Supplement 2). Significant main effects indicated habituation across and within sessions, independent of group (across session: *F*_5,7689.8_ = 21.21; *P* < .001; within session: *F*_2,7672_ = 9.31; *P* < .001). Yet, the group by within-session habituation interaction was not significant (*F*_2,7672.1_ = 0.87; *P* = .42). There was a significant main effect of discrete VR events (*F*_11,7667.6_ = 242.33; *P* < .001) and a significant interaction between group and reactivity to these events (*F*_11,7667.6_ = 2.19; *P* = .01), which indicates some VR events elicited greater reaction (eg, SC during driving vs an explosion), and the active tDCS group exhibited reduced reactivity. We did not observe correlations between changes in SC during tDCS plus VR and symptom change over the same period.

### Safety

Adverse effects were mild and consistent with the known safety profiles of tDCS and VR (eTable 3 in Supplement 2). The frequency of adverse effects did not differ between groups. Four serious adverse events occurred: 2 participants in the active tDCS group had exacerbations of chronic gastrointestinal illnesses, and 1 participant in the sham tDCS group had a syncopal episode. These were determined to be unrelated to study participation. One participant in the sham tDCS group had treatment-emergent suicidal ideation that was possibly related to participation.

## Discussion

This randomized clinical trial of tDCS plus VR for warzone-related PTSD demonstrated that 6 sessions of active tDCS plus VR over 2 to 3 weeks was superior to sham tDCS plus VR in improving PTSD symptom severity on the PCL-5, the measure most widely used in VA mental health services. Effects were nearly identical with those observed in our pilot study, clinically meaningful, and increased over time. Participants in the active tDCS group experienced enhanced psychophysiological habituation to VR warzone cues, as would be expected if the combined treatment facilitated learning and memory, replicating our pilot study findings.^26^ Additionally replicating our pilot results, PTSD symptoms continued to improve over the 1 month following active tDCS plus VR treatment. Three-month clinical outcomes did not reach significance, possibly due to attrition, as effect sizes were undiminished. Participants could not determine if they received active or sham stimulation. Adverse effects were mild and expected for the methods used.

These results should be placed in the context of an inexpensive and accessible technical setup and modest participant burden. This brief intervention is amenable to wide implementation, and ongoing development of these technologies might enable home therapeutic use. These methods also demonstrate an approach to controlling the context of brain stimulation, which is among the most difficult of factors to address in brain stimulation studies.

Observed symptom improvements were specific to PTSD, consistent with our mechanistic hypothesis. There were no meaningful changes in depression, and related effect sizes were modest. This is in contrast with prior studies of transcranial magnetic stimulation to a different brain target,^30,44^ in which changes in PTSD and depression occurred in parallel.

While there were no clear correlations between changes in SC and symptoms during tDCS plus VR procedures, as previously observed,^26^ symptom improvement continued after active tDCS plus VR. Changes in social and occupational functioning were delayed until the 3-month follow-up period. Speculatively, participants may have engaged in less avoidance over time, resulting in naturalistic exposures and positive reinforcement; this is an important topic for future studies with longer follow-up.

Participants receiving active tDCS to the VMPFC demonstrated significantly greater between-session habituation compared with the sham tDCS group, consistent with better reconsolidation or memory for safety learning. Most psychophysiological changes occurred early; if, as hypothesized, the psychophysiological changes reflect the learning processes underlying symptom improvement (ie, habituation), then extending the course of tDCS plus VR might be unlikely to provide additional benefit for individuals not showing physiological changes. Regardless, these and our prior results suggest that the relatively brief treatment course here is worth testing in a more definitive study. Of note, this intervention did not individually personalize the VR, as it was designed to incorporate broadly shared traumatic experiences. While this yielded standardized methods, whether individualized VR would improve outcomes remains unknown.

### Limitations

This study has some limitations. There was a large attrition rate at 1 and 3 months of follow-up, which hampers firm conclusions regarding longer-term effects. Additionally, many participants were using concurrent stable treatments, and the interaction between these and tDCS plus VR remains unknown. Participants had moderate symptom severity, and self-reported PTSD severity was included in stratified randomization. While electrical field modeling informed electrode placement,^45,46^ individualized modeling was not available at study inception and was not added to maintain consistency. All participants had prewarzone trauma, so we cannot disentangle whether that contributed to observed effects. By design this study used an augmentation approach and did not separately control for VR. The COVID-19 pandemic also significantly impacted recruitment, retention, and plausibly outcomes related to quality of life and social and occupational function (eAppendix 5 in Supplement 2).

## Conclusions

This randomized clinical trial replicates our pilot study^26^ and demonstrates the potential of combined tDCS and VR for PTSD treatment. Subjective results were accompanied by objective reductions in pathological arousal. This intervention imposed low participant burden, had modest technical cost, and yielded clinically meaningful improvements in an otherwise difficult-to-treat patient population. This reflects an important step forward in the use of combined brain stimulation and contextual control and underscores the innovative capability of these technologies.

## Notes

Supplement 1.
Trial Protocol and Statistical Analysis Plan

Supplement 2. **eTable 1.** Service-Connected Conditions **eTable 2.** Trauma Types **eTable 3.** Side Effects by tDCS Group **eFigure.** Methodological Overview **eAppendix 1.** Staff Blinding **eAppendix 2.** Randomization **eAppendix 3.** Cluster Analysis for Clinical Outcomes **eAppendix 4.** Psychophysiology **eAppendix 5.** Impact of COVID-19 Pandemic

Supplement 3.
Data Sharing Statement
