# Supplementary material for: Virtual Reality and Transcranial Direct Current Stimulation for Posttraumatic Stress Disorder: A Randomized Clinical Trial
Source: JAMA Psychiatry. 2024 Mar 6;81(5):437–46. doi: 10.1001/jamapsychiatry.2023.5661 (PMC10918574; doi:10.1001/jamapsychiatry.2023.5661)
Supplement: Supplement 1. — Trial Protocol and Statistical Analysis Plan [file jamapsychiatry-e235661-s001.pdf]

Combined Transcranial Direct Current Stimulation and Virtual Reality for PTSD

SPECIFIC AIMS

The purpose of the proposed study is to evaluate whether transcranial direct current stimulation (tDCS) can augment the effects of trauma-related virtual reality (VR) exposure as a treatment for Veterans with PTSD. This combination, which we call tDCS+VR, capitalizes on our experience with tDCS in PTSD and is designed to assess the efficacy of an adequately powered, randomized controlled study of tDCS+VR for PTSD that quantifies the relationship between changes in PTSD symptom severity, PTSD-relevant psychophysiology, and quality of life/social and occupational function (QOL/SOF).

The public health significance of PTSD cannot be overstated. PTSD is highly prevalent in Veterans, particularly Veterans from Operations Iraqi and Enduring Freedom and New Dawn (OIF/OEF/OND). PTSD is associated with significant psychiatric and medical comorbidity. Critically, PTSD prevents Veterans from reintegrating into society and is associated with poor quality of life including significant occupational and social dysfunction. Despite its prevalence and impact, the success of currently available treatments are mixed, highlighting the need for novel approaches that aim to reduce symptoms and improve functional outcomes.

Neurobiological models hypothesize that PTSD involves ineffective "top-down" modulation of exaggerated fear responses due, in part, to pathologically reduced activity in the brain's ventromedial prefrontal cortex (VMPFC). On this view, ineffective VMPFC regulation of fear responses prevents the generation of safety memories and allows PTSD symptoms to persist. Therefore, neuroscience-driven treatments for PTSD should focus on 1) improving safety learning, more commonly known as habituation, and 2) improving retention, or recall, of safety memories. Accordingly, targeted approaches to increase VMPFC activity have the potential to alleviate the core neurobiological deficits of PTSD.

Transcranial direct current stimulation (tDCS) holds considerable promise for this area of research. This approach to non-invasive brain stimulation uses very low electrical currents to increase or reduce the likelihood of neuronal firing (i.e., priming for depolarization or hyperpolarization). As such, tDCS readies the brain for subsequent responses to external stimuli to facilitate learning and memory. Moreover, tDCS has several advantages compared to other brain stimulation approaches, including a strong safety profile, ease of implementation, low expense, and an ability to target stimulation to the VMPFC. Because successful outcomes from exposure to trauma-related content (i.e., as used in exposure-based therapy for PTSD) require an adaptive learning process, applying tDCS in combination with exposure to trauma cues may effectively boost exposure-based learning. Our pilot research showed that tDCS can enhance safety memory formation in healthy volunteers and Veterans with PTSD, and demonstrated the clinical potential and technical feasibility of combined tDCS and exposure. However, the relationship between tDCS-augmented safety learning, PTSD symptom reduction and QOL/SOF has yet to be tested. Therefore, tDCS+VR provides an ideal standardized environment in which to test the efficacy of this combination. This evaluation is critical to determine real-world effectiveness of this treatment and will be assessed through the following Specific Aims:

Aim 1: Prospectively evaluate the efficacy of tDCS+VR in Veterans with chronic PTSD.

Challenge: To perform a randomized controlled trial, with adequate statistical power, to

test the efficacy of tDCS+VR to reduce symptoms of chronic PTSD (Aim 1A) and improve QOL/SOF (Aim 1B).
<u>Approach:</u> We will randomize 90 Veterans with chronic PTSD, and deliver sham-controlled tDCS to the VMPFC during a standardized 6-session VR exposure regimen. We will evaluate outcomes based on changes in PTSD symptoms (1A) and QOL/SOF (1B), comparing pretreatment baseline to endpoint, and at 1- and 3- months following study procedures.

Aim 2: Evaluate the relationship between psychophysiology, PTSD symptoms, and QOL/SOF.
Challenge: To test our hypothesis that tDCS-related changes in psychophysiological reactivity is correlated with PTSD clinical symptoms and QOL/SOF.
Approach: We will record skin conductance reactivity and compare baseline to endpoint, to correlate these measures and evaluate changes attributable to active tDCS+VR compared to sham.

Exploratory Aim 3: Explore the relationship between neuroimaging, PTSD and QOL/SOF.
Challenge: To explore our hypothesis that active tDCS improves the pathological neurocircuitry of PTSD.
Approach: We will use functional magnetic resonance imaging to measure resting state functional connectivity (RSFC), and evaluate whether active tDCS, compared to sham, increases RSFC between the VMPFC and amygdala. We will assess whether RSFC correlates with, or potentially predicts, changes in symptoms, psychophysiology and QOL/SOF.

Exploratory Aim 4: Explore the individualization of transcranial direct current stimulation intensity based on individual brain anatomy to inform future research.
Challenge: To explore whether variability in obtained electrical fields based on individual neuroanatomy can guide the implementation of a standardized tDCS dosage.
Approach: We will perform electrical field modeling to evaluate how neuroanatomy influences the amount of electrical current reaching target regions. This will allow determining, on an individual basis, the required tDCS intensity in order to obtain a prespecified electrical current in the brain. Future research will use this approach to standardize electrical current delivery.

BACKGROUND

A core component of PTSD is the inability to inhibit a maladaptive fear response (Rothbaum & Davis, 2003; VanElzakker et al., 2014). In real-world terms, this means Veterans struggle to overcome maladaptive fear learned during deployment, leading to significant psychosocial disability. To this end, exposure-based psychotherapy seeks to help Veterans learn that the hazardous experience (i.e., the cause of their PTSD) is no longer present or threatening in their current environment (Foa & Kozak, 1986; Foa et al., 2008). The extinction of conditioned fear, also called “extinction learning,” is a laboratory analogue for exposure therapy and investigates how the brain forms new safety-based memories (Milad & Quirk, 2012). Both processes, in the clinic and the laboratory, rely upon repeated exposure to a feared stimulus, but without occurrence of the feared consequences. The goal of this process is to form safety memories that inhibit and reduce fear associated with their original trauma or danger memories. If the new safety memory is sufficient, the individual can recall this safety memory, a process termed “extinction recall.” Abnormalities in extinction learning and recall are extensively associated

with PTSD (e.g., Milad et al., 2008; 2009), indicating that neuroscience research can be used to develop treatments for this disabling disorder.

During extinction learning, psychophysiological responses, such as fluctuations in skin conductance reactivity, are used to objectively assess sympathetic arousal associated with fear responses (Boucsein 2012). Heightened physiological responsivity during extinction learning and recall in PTSD (e.g., Milad et al., 2008; 2009) mimics the heightened responsivity to trauma-related cues (Pole 2007). Furthermore, reductions in physiological reactivity appear to be associated with therapeutic response (Blanchard et al. 2002; Griffin et al. 2012). Therefore psychophysiological assessments, derived from translational neuroscience, may potentially be used as objective indicators of treatment efficacy.

One related question is how to use lessons learned from neuroscience to improve clinical outcomes. There is an emerging understanding that emotional engagement in PTSD therapy is a crucial component of success (Foa, Huppert, & Cahill, 2006). One appealing approach to maximize emotional engagement is using immersive and contextually relevant virtual reality (VR) environments (Wiederhold & Rizzo 2005; Opris et al 2012). From a treatment development perspective, VR has the additional benefit of providing a standardized environment for hypothesis testing. This was recently demonstrated by Rothbaum et al., (2014), who tested whether VR could be augmented with pharmacotherapy (i.e., D-cycloserine) to accelerate extinction learning. While they did not find the hypothesized medication effect, they demonstrated a robust effect of VR. Their outcomes indicated that VR-based extinction could be successful, and that a different augmentation approach, informed by translational models and neuroimaging, might be more efficacious to improve symptoms and quality of life in Veterans suffering from PTSD.

The most consistent findings in animal models and human studies of PTSD are pathologically elevated activity in the amygdala and dorsal anterior cingulate cortex, which are regions that promote fear response. This is alongside reduced activity in the ventromedial prefrontal cortex (VMPFC), a region that suppresses fear response (Quirk et al., 2006; Etkin & Wager, 2007; Milad & Quik, 2012; VanElzakker et al., 2014; Koch et al., 2016). VMPFC activation during extinction learning predicts extinction success, and is associated with reduction of amygdala-driven fear expression (Rosenkranz et al., 2003; Phelps et al., 2004; Quirk et al., 2006). Supporting this model are results from studies of PTSD patients that showed reduced VMPFC activation during fear extinction, compared to controls (Rauch et al., 2003; Bremner et al., 2005; Shin et al., 2006; Milad et al., 2009; Rougemont-Bücking et al., 2011). Finding new methods to increase intrinsic VMPFC activity during safety learning may be a promising method to improve safety memory generation and retention, therefore leading to more effective PTSD treatments.

Non-invasive stimulation is an exciting and rapidly growing area of PTSD research. Prior work has largely focused on using repetitive transcranial magnetic stimulation (TMS) to reduce PTSD symptoms (reviewed in Karsen et al., 2014), including recent research from our own group (Philip et al., 2016a). Since PTSD is a disorder of brain circuits involved in emotional learning and memory, using brain stimulation during PTSD-relevant safety learning and memory generation (such as exposure to trauma-cues or context) may be an effective therapeutic approach (Baek et al., 2012; Marin & Milad, 2015). To date, the two preliminary studies of PTSD using TMS combined with exposure reported reduced clinical symptoms and psychophysiological arousal (Osuch et al., 2009; n = 9; delivered rTMS during exposure therapy, and Isserles et al. (2013); n = 10 per group; delivered deep TMS immediately following

exposure). Both papers highlight the difficulty of providing TMS and therapy at the same time. This is congruent with our clinical experience at the Providence VA Neuromodulation clinic. We have attempted to provide psychotherapy during clinical TMS, and Veterans informed us that it was difficult to focus due to discomfort and distraction from the TMS pulses. Therefore, stimulation that can be easily combined with psychotherapeutic modalities represents the important next step to develop a neuroscience-informed rehabilitation treatment for Veterans with PTSD.

Transcranial direct current stimulation (tDCS) is another approach to non-invasive brain stimulation. tDCS alters cortical excitability via subthreshold modulation of neuronal resting membrane potentials using a weak constant electrical current (Nitsche et al., 2008). Effects of tDCS are dependent upon the current polarity, where positive current flow (i.e., anodal stimulation) increases the likelihood of neuronal depolarization, whereas negative current flow (i.e. cathodal stimulation) decreases the likelihood of action potentials. Stimulation is typically provided over a 20-30 minute period, using a very low direct current (1-2mA), to change neuronal cell membrane potentials. As such, tDCS readies the brain for subsequent responses to external stimuli to facilitate learning and memory (Coffman et al., 2014).

Prior work has demonstrated that tDCS in the range of 1-2 mA can modify fear memories that correspond to the direction of stimulation. Asthana et al. (2013) showed that inhibitory cathodal tDCS to the prefrontal cortex in healthy controls could reduce fear memory consolidation, and Mungee et al., (2014) demonstrated excitatory anodal tDCS, delivered to the prefrontal cortex could enhance fear memory (in an experiment designed to increase fear). In another study, Ironside et al., (2016) reported that tDCS reduced threat vigilance in healthy controls. Although these studies indicate that prefrontal tDCS can change fear memory processes, more clinically relevant use would be excitatory anodal tDCS delivered during fear extinction to brain regions involved in fear extinction (i.e. to accelerate extinction or increase safety memory)(Marin & Milad, 2015). Work from our group indicates that this is possible. We reported that excitatory tDCS targeting the VMPFC during fear extinction improves extinction in healthy controls (van 't Wout et al., 2016), and tDCS to the VMPFC following extinction learning may boost extinction memory in Veterans with PTSD (van 't Wout, Philip et al., 2017).

STUDY OUTLINE

The goals of this proposal are to: 1) prospectively evaluate effectiveness of active tDCS versus sham tDCS, combined with VR trauma cue-based exposure, on PTSD symptom improvement and quality of life/social and occupational function (QOL/SOF); 2) assess how psychophysiological arousal correlate with clinical symptoms and QOL/SOF; and explore both 3) whether changes in neural circuitry correlate with PTSD symptoms and QOL/SOF, and 4) methods to standardize tDCS intensity based on individual neuroimaging. To do this, we will randomize 90 Veterans with PTSD from the Providence VAMC. Forty-five participants will be randomly assigned to receive active tDCS+VR, and 45 will receive sham stimulation+VR. All participants will receive a total of six VR sessions over the course of about two to three weeks. These procedures are modeled after the VR-based exposure therapy protocol by Difede and Hoffman (2002), Rothbaum et al., (2014), and experience gathered from our pilot data (van 't Wout et al., 2016).

STUDY POPULATION

We will recruit OIF/OEF/OND Veterans with current warzone-related PTSD. The study population will consist of Veterans between 18 and 70 years old. They will be men or women in the greater Providence and Boston areas who have been discharged from active duty, or who served in the National Guard or military reserve units. We will recruit a sample of OEF/OIF/OND Veterans from the Providence VAMC PTSD, mental health, and primary care clinics, with demographic features consistent with the population we treat.

<u>Sample size:</u> We propose to randomize 90 participants, 45 participants will be randomized to receive active tDCS during exposure to war-related scenes within the VR system, and 45 participants will receive be randomized to receive sham tDCS during exposure to war-related scenes within the VR system. In order to achieve randomization of 90 individuals, we anticipate enrollment of up to 120 individuals. Please note that recruitment will cease once randomization goals are met (i.e., n=~45 Veterans per condition).

Power Analysis: This study is designed to have adequate power to detect differences between active and sham tDCS for its Primary Aim. Minimum detectable standardized effect size (d) computations are simplified using Lehr's equation (Lehr, 1992) given a known per-group sample size (n) and using a type-I and type-II error rate of 5 and 20%, respectively, using $d = \sqrt{(16/n)}$. Under an ANCOVA framework, the modification $\sqrt{16(1\text{-}r2)/n}$ where r is the pre-post correlation of the outcome variable can be used. We propose data analysis on all 45 participants per group with an adjustment in the effect size to account for the assumption of null effects due to missing data. For Aim 1, in our prior studies of non-invasive brain stimulation for PTSD (Philip et al., 2016a+b), we observed a pre-post correlation of .35 for the PCL. Therefore, we expect to have adequate power to detect an effect size (Cohen's d) of .56 or larger (n=45/group, autocorrelation = .35), which is a medium standardized effect size (Cohen, 1988) given our design and planned analysis. This minimally detectable effect falls within the range of treatment effect sizes reported in the literature on the PCL with d= .59 (Forbes et al., 2001) and psychotherapy for PTSD with d= .52 immediately after treatment, and d= .64 at 3-14 month follow-up, and d= .58 on hyperarousal (relevant for Aim 2)(Sherman, 1998). In a meta-analysis, Opris et al. (2012) reported similar effect sizes for VRET as compared to cognitive and/or behavioral or in vivo exposure, and Rothbaum et al. 2014, reported a large effect size (d=1.56) associated with a similar VR paradigm as used in this proposal. Data from our pilot (N=10) demonstrates an effect size of d=.74 for tDCS+VR vs. sham on PTSD symptom severity at follow-up and effect size of d= 1.68 for a change in psychophysiological arousal for tDCS+VR. While we recognize these effect sizes are likely inflated due to the small sample size, they demonstrate effects in correct direction To estimate potential changes in VMPFC-BLA functional connectivity, pilot resting state data was acquired from 25 participants both before and after rTMS (i.e., 50 observations) for chronic PTSD (Philip et al., 2016b). The average effect size in standard deviation units (analogous to Cohen's d) of the increase in VMPFC-BLA connectivity across treatment sessions was large (Cohen's d= 1.08), indicating that the larger sample size in this study will be sufficient for Exploratory Aim 3.

<u>Inclusion/exclusion criteria:</u> The inclusion and exclusion criteria were formulated with the intent of being as unrestrictive as possible, while still ensuring the safety of participants and maintaining the internal validity of the study.

> *Inclusion criteria:* Principle inclusion criteria will be symptoms of chronic PTSD, meeting DSM-5 criteria and with trauma related to warzone experience. Eligible male and female Veterans will be between ages 18-70, and, if in treatment, symptomatic despite ongoing stable treatment regimens for at least 6 weeks prior to study procedures.

Ongoing medications and psychotherapy will be allowed to continue unchanged during the study. Veterans must also be willing and able to comply with all study related procedures and visits, and capable of independently reading and understanding study materials and providing informed consent.

Exclusion Criteria: For safety, participants must meet established screening criteria safety during MRI, as MRI procedures are a component of the study. These measures prohibit (but are limited to) the following: Cardiac pacemaker, implanted device (deep brain stimulation) or metal in the brain, cervical spinal cord, or upper thoracic spinal cord. Additional tDCS-specific exclusions are: skin lesions at the site of stimulation that may increase conductance (e.g., vascular moles or angiomas); pregnancy/lactation, or planning to become pregnant during the study; lifetime history of moderate or severe traumatic brain injury (TBI); current unstable medical conditions; current (or past if appropriate) significant neurological disorder, or lifetime history of a) seizure disorder b) primary or secondary CNS tumors c) stroke or d) cerebral aneurysm. Other exclusions are any primary psychotic disorder, bipolar I disorder, active greater than moderate substance use disorders (within the last month, excluding nicotine/caffeine, assessed with urine drug testing if appropriate), have active suicidal intent or plan as detected on screening instruments or in the investigative team's judgment is likely to attempt suicide within 6 months, and other conditions or circumstances that, in the opinion of the investigator team, have the potential to prevent completion and/or have a confounding effect on outcome assessments.

Concurrent Treatment: Medications and Psychotherapy**:** The combination of tDCS+VR, psychophysiology, neuroimaging, and ongoing treatment for PTSD adds a degree of complexity to the current study. An ideal study design would be to include treatment-free participants so that effects observed could be solely attributed to tDCS+VR. However, we are most interested in recruiting Veterans that reflect patients receiving VA care to ensure the generalizability of our results. Our prior experience conducting neuromodulation studies with medication free depressed patients (Philip et al., 2016c) confirmed that recruitment and retention of treatment-free patients is highly challenging and would endanger the feasibility of the current proposal. Furthermore, removal of medications and/or psychotherapy from clinically ill Veterans with PTSD raises significant ethical concerns. Therefore, we will follow the design of many prior studies that examine effects of neuromodulation delivered as an adjunct to stable pharmacotherapy and/or psychotherapy. We will ask participants to continue therapy unchanged during their participation, and require that psychopharmacologic medications be stable for at least 6 weeks prior to participation. Participants will be encouraged not to change medications unless medically indicated, and based on our experience we anticipate these changes to be very modest. If they occur, they will be recorded and explored using a sensitivity approach during data analyses, and we will explore outcome differences between participants receiving concurrent treatment (i.e., (medications and/or therapy) versus those who are not receiving concurrent treatment.

PROTOCOL DESIGN/RESEARCH PLAN

Recruitment Procedures: Participants will be recruited from the Providence VA Medical Center (only Veterans registered with the PVAMC prior to enrollment will be included in this study), through VA-approved resources, e.g. internet resources -twitter, Facebook-, and referral lists from the OEF/OIF/OND program to use for screening prior to enrollment. This will help identify potential participants for screening in CPRS and to whom we will send recruitment

letters by mail. Brochures will be distributed and copies of brochures for patients will be available at registration, primary care, and at mental health clinics. Brochures and flyers will be posted on bulletin boards located around the medical center.

Brochures will be displayed in the Rhode Island area VA Medical Centers and Veterans Centers, as well as in local primary care clinics and in other community locations and events likely to be visited by Veterans and their families. Brochures will also be distributed at the Providence VA Medical Center to veterans seeking treatment. The study will be explained to VA staff and providers at these locations and they will be encouraged to mention the study and present the brochure. A representative of the study (i.e., study coordinator or research assistant) will periodically be available on-site at primary care and mental health clinics serving OEF/OIF veterans at the Providence VA Medical Center. This representative will be available to answer questions and provide information about the study.

We will also review medical records in CPRS to identify patients who seem eligible for the study. If we find a potentially eligible person through the review of medical records, study staff will send the patient an official letter explaining the study and indicating that a specific research staff member will contact the patient on a specific day about the study. Staff will call the patient on the designated date.

We will also advertise using IRB approved language through the IRB approved brochure language, fliers or PowerPoint advertisement (for VA monitors) posted throughout PVAMC, the CBOCs, and Vet Centers. We will issue press-releases to local RI and Southeastern Massachusetts news outlets and the PVAMC Research Service website. We may also advertise through the PVAMC Research Newsletter, Veteran Associated Newsletters and magazines and local newspapers. Please refer to Appendix A for recruitment language.

Patient name, last four digits of the social security number, date of birth, telephone number, provider, date/time of next appointment with provider, and contact history will be recorded in a recruitment log once the patient has been identified as eligible. This information is necessary for tracking recruitment and contacting patients.

<u>Subject Pre-Screening</u>: Potential participants will enter the screening phase. Screening information will be obtained over the phone and structured so as to minimize participant burden. Staff will collect identifiable and/or protected health information about the participant including name, date of birth, telephone number, address, dates and locations of military deployments, medications, and medical and psychological conditions. Information will be supplemented with relevant information from CRPS (e.g. medication doses, diagnoses, etc.). Research staff will also ask about information that includes 38 USC 7332 data and, specifically, information on alcohol and drug use. Potential participants who will answer affirmatively to current abuse of alcohol and drugs will be excluded from the study. Special protections are in place to protect this information on alcohol and drug use. Women of childbearing age will also be inquired about pregnancy because of precautionary concerns about pregnant women receiving tDCS. When appropriate, participants will undergo pregnancy testing prior to study procedures.

Contact information will be recorded in a password protected file and stored in a restricted folder on the secure Providence VA research server. For potential participants who are found ineligible at pre-screening or elect not to participate, pre-screening responses will be physically separated from any identifying information and all cross links will be removed so that it cannot

be re-identified. Protected health information collected during the pre-screening process will not be linked to those participants who are either ineligible or elect not to participate.

Description of the Informed Consent Process: All potential participants will be asked to fill out informed consent. Research personnel will review all parts of the consent form with participants, including the description of study assessments and protocol, limits to confidentiality, alternatives to participation, and potential risks and benefits, as well as the right to withdraw from the study without penalty. Participants will be informed that their involvement in this study, or refusal to participate, will not affect their treatment with the VA. Participants will be given contact numbers for PI and for the IRB. The PI will be available to answer any questions or provide additional information. Participants may be asked to sign releases of information to their treating physician to gather additional information for this study.

Potential participants will be given as much time as they need to review the consent form, to ask questions as needed, and to make a decision as to whether or not to participate, as long as they continue to meet the study eligibility criteria. Potential participants will have the option of taking the study consent form and HIPAA authorization home to read further and think about whether or not they want to be involved in the study, and/or talk about the study with family, friends or caregivers if they choose. There is no time limit for this decision-making process, as long as recruitment is continuing and they continue to meet criteria for the study. A complete description of the study and full disclosure of the potential benefits, risks and alternatives will be provided. Once a potential participant decides they want to enroll in the study, he will be required to sign a consent form prior to participation in the study. A copy of the signed form will be given to participants to take with them. The original signed consent form will be kept in the principal investigator's study file and a copy will be placed in the Veterans' medical record. Participants will be told that they may refuse participation without any negative consequences, and that if they decide to participate, they will be free to withdraw from the study at any time.

Baseline/Screening: After consenting to study procedures, eligible participants will complete a baseline assessment at the Providence VA Medical Center. All participants will be asked to complete a battery of self-report assessments and baseline interviews (see section S*tandardized Measures and Participant Assessments*). Age, demographics, race, education, current treatments and psychopharmacologic medication history will be recorded. The Mini International Neuropsychiatric Interview for DSM-5 (Sheehan et al., 1998) may be used to establish psychiatric diagnoses, current and lifetime, and inclusion/exclusion criteria. When appropriate, participants will be asked to undergo a pregnancy test or urine drug test per the previously noted exclusion criteria. In addition, study staff will perform tDCS safety screening which also includes assessment of the skin on the participant's scalp before each tDCS session where the electrodes will be placed to assure there are no lesions, cuts, or exclusionary skin disorders prior to tDCS. Also participants will be MRI safety screened before each MRI.

This first baseline/screening visit will take approximately 3-3.5 hours. The Principal Investigator will make the ultimate decision of whether or not the participant is eligible to participate in the study. Participants that do not meet the inclusion criteria will be informed that, based on the information collected, it has been determined that they are not appropriate for the current study. Appropriate referrals to other health care providers at the VA or to outside providers if desired by the participant, will be made when indicated.

Procedures:

Visit Schedule: Study participation involves a total of up to thirteen visits at the Providence VA Medical Center and/or the Brown University MRI Research Facility. After the baseline visit (visit 1), Veterans will undergo pre tDCS+VR MRI (visit 2). Veterans will then attend six active or sham tDCS+VR sessions (visits 3-8) delivered over about 10-15 business days, with at least 24 hours in between sessions. Each session will last approximately 60 minutes inclusive of study set up, tDCS+VR and post-session debriefing. MRI imaging data will be acquired after the completion of all sessions (visit 9). Veterans will return to the PVAMC one and three-months later for post-treatment follow-up assessments (visits 10 & 11). Participants will have the option of participating in an additional visit (visit 12) that will include an EEG. This visit will take place before tDCS+VR sessions. Eligible participants will have the option to participate in an additional MRI visit (visit 13) around the 1-month follow-up period. Procedures for each study component are described subsequently.

If the study PIs deem it appropriate or necessary, study visits/assessments that can occur virtually may be conducted via video (VA approved VA Video Connect) or telephone.

Randomization: Following eligibility determination, participants will be randomized to either active tDCS+VR or sham stimulation+VR, using urn randomization (Wei & Lachin, 1988) with mild TBI, sex, and depression diagnosis (yes/no) as blocking variables (i.e., variables of interest and/or could impact outcomes).

MRI: Study staff will work with the participant and the MRI staff to schedule the MRI (additional MRI sessions may occur in the case that the participant needs to be rescanned due to MRI equipment failure). All MRIs will be performed on the PVAMC MRI Siemens Verio™ 3.0 Tesla scanner, if needed the MRI scans will be done at the Brown MRI Research facility. All images will be obtained during a 45-minute scan session. The first 15 minutes will be used to familiarize the participant with imaging procedures, assess for pregnancy if indicated, discuss any changes in mood, sleep, and/or medication since their last visit, and participants will be asked to complete the Perceived Stress Scale prior to entering the scanner. Once in the magnet, research staff will ensure that the participant is comfortable, able to view the task screen, and has been provided the necessary instructions regarding communication with research staff during the scan (e.g., using emergency squeeze ball). This will be followed by acquisition of high-resolution anatomic images (MEMPRAGE), functional multiband echoplanar (MB-EPI) images, and diffusion imaging using a 64 channel head coil. Freesurfer-preferred multiecho MEMPRAGE protocol will capture anatomic images (e.g., FOV: 256 mm, 176 sagittal slices, slice thickness 1 mm, phase encoding anterior-posterior; TR 20ms, TE 1.85+n2.0 ms (where n = 0,..,7)). Twelve minutes of resting state data will be obtained while participants view a light fixation cross against a black background, following MB-EPI methods used in the Human Connectome Project (e.g. TR = 720 ms, TE = 33.1 ms, FOV = 280mm, 2mm isotropic, potentially followed by a separate 12 minute scan inclusive of two runs utilizing opposite phase encoding to minimize potential distortion). About eight minutes of diffusion imaging may also be used to evaluate network architecture (e.g. TR = 3600 ms, TE = 71.6 ms, FOV = 208 mm, slice thickness 1mm). After each scan, the participant will be queried about their experience in the scanner to assess for anxiety and/or discomfort. *Participants deemed MRI compatible/eligible will be given the option of undergoing an additional MRI during the follow-up period (e.g., at/around 1-month). The follow-up scan is completely optional and does not affect the participants ability to participate in the other parts of the study.*

MRI Preprocessing and Head Motion. We will use the Human Connectome Project (HCP) Minimal Processing Pipeline, which incorporates routines from FSL and Freesurfer for structural and functional preprocessing. We will utilize the Artifact Detection Tool (ART, implemented within CONN (Whitfield-Gabrieli & Nieto-Castanon, 2012) to reduce the impact of motion-related artifacts and non-neuronal signal on functional connectivity estimates (Power et al., 2011; Satterthwaite et al., 2013; Ciric et al., 2017). We will use principle component analysis to model noise in BOLD time series extracted from the white matter and ventricles using anatomical CompCor (aCompCor; Behzadi et al., 2007). aCompCor is an effective method for removing motion-related artifact, and does not involve global signal regression (Ciric et al., 2017), since global signal regression complicates the interpretation of resting state correlations and increases distance-dependent effects of motion (Murphy et al., 2009; Murphy and Fox, 2016; Ciric et al., 2017). Five principal components from each aCompCor compartment will be used in subject-level confound regression models, along with six motion parameters (with their first temporal derivatives) estimated during realignment. We will include high motion (>0.5 mm) or global signal variance (>3 SD) time points in confound regression models to further limit spurious variance. Residual effects of motion-related artifact will be quantified by measuring correlations between frame-to-frame root mean square frame-wise displacement and averaged connectivity estimates (Ciric et al., 2017). We considered other approaches for motion/artifact correction, and implemented aCompCor because it does not require a study-specific training dataset (required for motion correction procedures utilized in the Human Connectome Project, i.e., FIX ICA), and its superior ability to remove motion from resting state data (Ciric et al, 2017).

tDCS+VR:

tDCS Procedures

Device: Stimulation will be delivered using a Neuroconn DC-STIMULATOR PLUS (NeuroConn Inc, Ilmenau, Germany), which is on-site at the Center for Neurorestoration and Neurotechnology at the Providence VAMC and used in our pilot data (e.g., van 't Wout, 2016; van 't Wout, Philip et al., 2017). This device has built in safety features, such as impedance assessment and automatic cut offs and the ability to deliver double-blind stimulation. In addition, the device will be battery-driven to function as a constant current stimulator with a maximum output in the miliAmps range. Battery-driven operation, instead of electrical outlets, is necessary to power the device, as a potential malfunctioning device might deliver large intensities of electrical currents with no warning when powered by electrical outlets. The risk of suddenly high-intensity electrical current is absent since the device is powered by a 9V battery. We have compiled a picture of all the necessary materials that we will use for tDCS, see Figure 1A below.

Electrode Montage and Target Modeling: Electrode placement (i.e., montage) will follow the locations used in our pilot studies. We will use a 1 (anode) x 1 (cathode) unilateral electrode set-up (Nasseri et al., 2015). Following our Specific Aims, we will target the VMPFC. To determine the ideal stimulation montage, we performed current distribution modeling using tDCS Explore neurotargeting software by Soterix Medical (v. 4.0), New York, NY). Electrical field modeling indicated that VMPFC stimulation best occurs with the anode placed over EEG coordinates AF7/Fp1/AF3 (using the 10-20 EEG convention) and the cathode between EEG coordinate OZ and the contralateral mastoid (covering approximately PO8/P8).(see Figure 1B below). This placement mitigates undesired

nonspecific stimulation that might interfere with desired effects (e.g., observed in Abend et al. (2016) that used larger electrodes and different montage).

Stimulation Procedures and Parameters: Informed by our pilot experience, we will assess tolerability of tDCS for all participants by applying brief stimulation (1 mA for 30 seconds, with a ramp up/down over 30 seconds each) prior to VR. We found doing this mitigates participant dropout due to potential discomfort during the initial tDCS+VR experience. Prior to stimulation, staff with experience in tDCS will lightly clean the participant's head, and electrode sponges will be attached to the scalp using a rubber headband. For active tDCS, we will stimulate at 2mA for 25 minutes during VR. Each electrode will be placed in a 3x3 cm (9 cm^2) reusable sponge pocket saturated with 0.9% normal saline, resulting in current density of 2.22 A/m^2. This intensity will correct for stimulation intensity loss attributable to distant electrode placement (Moliadze, Antal, Paulus, 2010). Sham tDCS will include a 30-second ramp up to 1mA, 30 seconds of stimulation at 1 mA, followed by a 30-second ramp down to off. This procedure allows for detection of tingling on the skin associated with active stimulation in the beginning of the session, making participants less aware that they are receiving sham stimulation. In this double-blind design, participants will either receive active tDCS or sham during VR-session. In order to monitor the effectiveness of the blind, participants will be asked to guess which treatment they received after completion of all six VR-sessions. In addition, participants will be asked about tolerability of the tDCS after each VR-session.

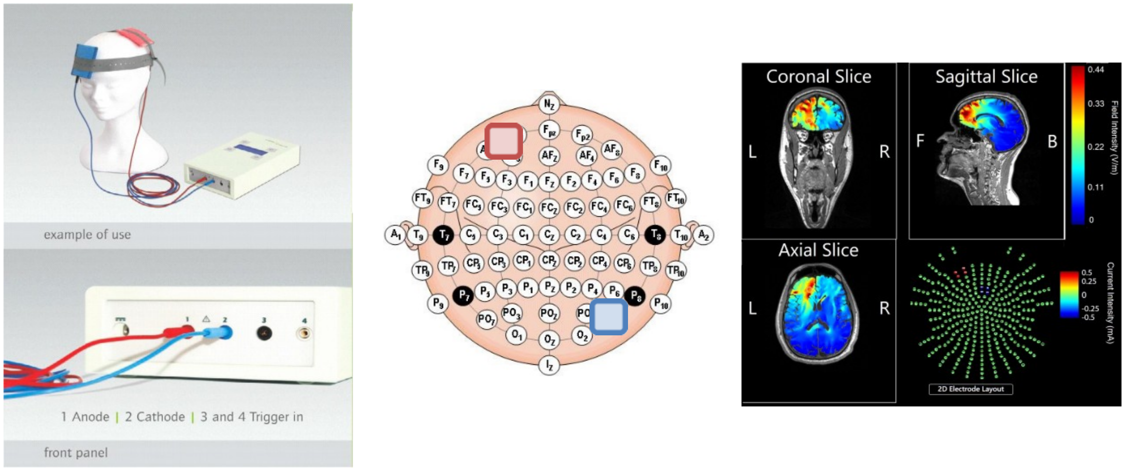

Figure 1A. Example of the to-be-used equipment for tDCS administration. 1B. 10-20 EEG system for reference and modeling.

VR Procedures: Participants will be presented with a standardized continuous driving scenario that matches their trauma location during deployment (i.e. Iraq or Afghanistan) based on schedule of VR administered in Difede and Hoffman (2002) and informed by Rothbaum et al., 2014. As in our pilot work, we will systematically start with a low intensity VR experience, and sequentially increase the intensity using Bravemind stimulus elements. In addition, participants will repeat the same VR scenario three times within one session to optimize desensitization. Participants will be asked to treat the scenes as if they were real, and provided safe words to stop the program at any time.

All participants will be presented with the same combat-related events (identical stimuli presented in Iraq or Afghanistan background environments), interleaved with 2-minute

continuous driving periods during which no other stimuli are presented (i.e., to control for psychophysiology). With each scene, the intensity of the experience will increase. The following is an example of scenes that may be used. In the first VR scene, participants will be presented with only the low intensity continuous driving scenario (i.e., participant riding in Humvee with background sounds) for two minutes. The second scene will increase in intensity by having the participant driving the Humvee, with the addition of an insurgent attack. The third scene will include driving, the insurgent attack, and an IED explosion. The fourth will include driving, insurgent attack, IED explosion, and explosion of a Humvee in front of the participant's vehicle. The fifth and final scene will include driving, insurgent attack, IED explosion, Humvee explosion, bridge ambush, and the smell of weapons fire. This series (or a series of similar scenes), of escalating intensity VR is repeated 3 times to allow within-session habituation. The total VR time is 24 minutes. Following the end of the VR protocol or termination of the procedure at the request of the participant or researcher, the researcher will remove the VR and the psychophysiological recording equipment. The participant will then be debriefed about their experience for safety.

VR program: We will use Bravemind VR (v. 1.0.4 or higher) from Virtually Better (Decatur, GA). The Bravemind system is an all-inclusive package that allows full immersion into virtual Iraq/Afghanistan using a head-mounted display and headphones. The system simulates the experience of commanding a light infantry company and a previous version of this package (Full Spectrum Command) has been used in prior studies at Providence VAMC (Spofford et al., submitted). The system was developed through collaboration between the Institute for Creative Technologies (ICT); entertainment software companies; the U.S. Army Training and Doctrine Command (TRADOC); and the Research, Development, and Engineering Command, Simulation Technology Center (RDECOM STC).

Psychophysiology: Psychophysiological measurements will be taken throughout the administration of the VR task. Heart rate and skin resistance are the primary measures that will be monitored during the VR protocol. Skin conductance will be recorded for 2 minutes prior to the VR session to establish a physiologic baseline, and throughout VR. Skin conductance reactivity to VR events will be measured by the phasic responses that occur after the presentation of discreet VR events following previous guidelines (Milad et al., 2005). The mean of the lowest baseline portion will be used to define tonic level. Heart rate will be calculated as beats per minute and will be averaged for each aim of the experimental procedure. The maximal calculated heart rate for each patient will be calculated by using the formula outlined in Wasserman et al. (1999): maximum heart rate (beats/min) = 220 minus participant's age in years. Each participant's heart rate will be monitored and the procedure will be stopped by the research staff if the participant's heart rate reaches this rate. The Biopac system and its devices are intended for physiological research purposes, and is designed to satisfy certain applicable test standards. It does not have FDA approval, as it is not a clinical, medical device intended for diagnosis, mitigation, treatment, or prevention of disease.

An IRM workstation that has Biopac data acquisition system software installed on it will be used to collect psychophysiological data. The electrocardiogram and galvanic skin response modules will be utilized for collection of heart rate and skin conductance, respectively. The subject will have 2 electrodes placed on his/her body to assess his/her heart rate. One electrode will be placed on the subject's upper right chest and one electrode will be placed on the left side of the subject's lower belly. Skin conductance will be measured using a constant 0.5 V through 8 mm Ag/AgCl

electrodes. Electrodes will be pre-filled with conductance paste from Biopac and placed on the palm of the participant's right hand. The Biopac conductance paste is an isotonic electrode paste specially formulated with 0.5% saline in a neutral base. The participant will wear the psychophysiological devices through their participation in each of the VR assessment sessions.

Electroencephalography (EEG; optional): Participants determined to be eligible will be given the option of participating in an electroencephalography (EEG) recording session before beginning the tDCS+VR sessions. Resting state EEG recordings will be collected with a 32- or 64-channel lead EEG cap, using the eego ANT Neuro system (inclusive of software and computer components). The session will last approximately 1 hour (this includes the set-up, orientation, and EEG recording). During the session, participants will be asked to sit quietly with their eyes open or closed. This visit is completely optional and is not required to participate in the previously mentioned study procedures.

<u>Standardized Measures and Participant Assessments:</u>

Abbrev:	Assessment Title:	Administered at:
**MINI**	Mini International Neuropsychiatric for DSM-5	BL
**CAPS-5**	Clinician Administered PTSD Scale	BL, EOT, PT
**PCL-5**	PTSD Checklist	BL, MP, EOT, PT
**LEC-5**	Life Event Checklist	BL
**CES**	Combat Experiences Scale	BL
**DES**	Dissociative Experiences Scales	BL
**IDSSR**	Inventory of Depressive Symptoms Self Report	BL, MP, EOT, PT
**PANAS**	Positive and Negative Affect Schedule	All VR sessions
**DAR**	Dimensions of Anger Reactions	BL, MP, EOT, PT
**PSQI**	Pittsburgh Sleep Quality Index	BL, MP, EOT, PT
**tDCS SE**	tDCS Side Effects Form	All VR Sessions
**STAI**	State-Trait Anxiety Scale	BL, EOT, PT
**Q-LES-Q-SF**	Quality of Life Enjoyment and Satisfaction Questionnaire	BL, EOT, PT
**SOFAS**	Social and Occupational Functioning Scale	BL, EOT, PT
**CGI-S**	Clinical Global Impressions – Severity	BL, EOT, PT
**CGI-I**	Clinical Global Impressions – Improvement	BL, EOT, PT
**Tx SF**	Treatment Satisfaction Form	EOT, PT
**Blinding**	Blinding	EOT

In the table above: BL, Baseline; MP, midpoint; EOT, end of treatment; PT, 1- and 3-month visits post treatment.

Mini International Neuropsychiatric Interview for DSM-5 (MINI): The MINI (Sheehan et al., 1998) will be used to establish psychiatric diagnoses, current and lifetime, and inclusion/exclusion criteria. Administration takes approximately 20-30 minutes.

Clinician-Administered PTSD Scale (CAPS-5): The CAPS (Blake et al., 1995) is the "gold-standard" structured clinical interview for PTSD assessment. It provides a continuous score of frequency and severity for each symptom and a dichotomous diagnosis of PTSD. A behaviorally-anchored probe question is provided for each symptom to increase the reliability of

administration. The CAPS has been found to have a sensitivity of .81 and a specificity of .95. It will be used to establish a PTSD diagnosis at baseline and change in scores over time will capture symptom change after tDCS and at the end of the follow up period. Administration takes approximately 60 minutes.

PTSD Checklist (PCL-5): The PCL-5 (Weathers et al., 2013b) is a 20-item self-report questionnaire, corresponding to the DSM-5 symptom criteria for PTSD. The PCL-5 asks respondents to identify the worst event that currently bothers them the most and then asks them to answer 20 questions related to the event. The items are rated on a 4 -point severity scales (0 = "not at all bothered by" to 4 = "extremely bothered ").

Life Event Checklist (LEC-5): The LEC-5 (Weathers et al., 2013c) assesses for lifetime trauma exposure.

Combat Experiences Scale (CES): This scale was developed specifically to assess trauma exposure in Iraq and Afghanistan veterans, but has also been used on veterans of various war eras (Hoge et al., 2004). As such, it includes items more specific to these wars (e.g. IEDs, searching homes). Items assess frequency of being in serious danger, number of firefights, injury including head injury, and frequency of exposure to 13 combat and other trauma events, and is rated on a scale from 0 (never) to 4 (10 or more times).

Dissociative Experiences Scales (DES): The DES-II is a screening tool for dissociative experiences and measures a wide variety of types of dissociation, including both problematic dissociative experiences and normal dissociative experiences (e.g., day-dreaming) (Carlson & Putnam, 1993).

Inventory of Depressive Symptoms (IDSSR): The IDSSR (Rush et al., 2013) is a detailed inventory of depressive symptoms developed to focus especially on symptom frequency. It is a self-report scale that provides a continuous measure of depression severity.

Positive and Negative Affect Scale (PANAS): The PANAS is a 20-item self-report questionnaire that is comprised of two mood scales, one measuring positive affect and the other measuring negative affect. Each item is rated on a 5-point scale ranging from 1 (“very slightly or not at all”) to 5 (“extremely”), to indicate the extent to which the respondent feels this way at the moment (Watson et al., 1988).

Dimensions of Anger Reactions (DAR): The DAR (Forbes et al., 2004) will be used to evaluate anger and related symptoms.

Pittsburgh Sleep Quality Inventory (PSQI): The PSQI is a self-report questionnaire that assessed sleep quality over a 1 month time interval. This will be utilized to assess changes in quality of sleep throughout the course of the TAVRE procedure.

tDCS side effects questionnaire: This questionnaire asks about the experience of more and less frequent occurring experiences during and after tDCS application. The questionnaire aims to assess whether participants experienced these symptoms giving insight into the tolerability and potential problems when implementing tDCS during treatment in the future.

State-Trait Anxiety Scale (STAI): The STAI (Spielberger et al., 1988) will evaluate changes in anxiety outside of PTSD symptoms.

Quality of Life Enjoyment and Satisfaction Questionnaire (Q-LES-Q-SF): The Q-LES-Q-SF (Ritsner et al., 2005) evaluates QOL and other areas of change related to functioning outside of symptom domains.

Social and Occupational Functioning Scale (SOFAS): The SOFAS (Morosini et al., 2000) will evaluate changes in functional status attributable to study procedures.

Clinical Global Impressions – Severity/Improvement (CGI-S/I): Clinical global impressions of severity and improvement (Guy, 1978) will be used to capture overall symptom severity and improvement associated with treatment.

Treatment Satisfaction Form – The treatment satisfaction form (Blatt et al., 1998) will gather feedback from participants related to study participation. This form is a five item self-report questionnaire to assess the participant experiences related to receiving tDCS+VR. In particular, this questionnaire aims to assess whether the participant notices any differences after stimulation as compared to before stimulation with respect to their condition and feelings, as well as provides an opportunity to express aspects that may have been problematic.

Blinding – At participation endpoint Veterans will be queried whether they received active or sham stimulation.

<u>Additional Treatment:</u> Participants are allowed to pursue any medical or mental health treatments while enrolled in the study.

<u>Monetary Considerations</u>: Participants will be compensated with up to $425, using electronic funds transfer (ETF) or VA-approved gift cards (e.g. CVS), for participating in this study. A prorated system of payment will be used. Participants will be paid $75 when they complete the first baseline study visit, $75 after completing the 6 virtual reality sessions, $75 after the 1 month follow-up, and another $75 after the completion of the final follow-up visit 3 months later. If participants are unable to finish the study completely, they will still be paid for their time based on the amount of the study completed. If participants chose to participate in the optional EEG visit, they will be compensated with $50 using EFT or VA approved gift cards. If MRI compatible participants choose to participate in the optional follow-up MRI, they will be compensated with $75 using EFT or VA approved gift cards.

<u>Subject Retention</u>*:* Permission will be requested at the time of informed consent to obtain the name and phone number of a close relative, friend, or other person who is likely to maintain contact with the participant, and to contact that person if attempts to contact the participant are unsuccessful. On the last day of tDCS+VR, appointments will be made for the one- and three-month follow-up assessments. Participants will receive a letter one week prior to the follow-up appointment as well as a reminder call 3- and 1 days prior. Participants who fail to appear for a scheduled assessment will be contacted by phone or mail when necessary for rescheduling, and five contact attempts will be made before a participant is considered unreachable. If participants move away during study participation, or are otherwise unavailable for an in-person assessment, we will perform follow-up assessments over the telephone or by VVC.

DATA ANALYSIS

General Statistical Approach and Considerations: Random assignment of participants with regard to baseline characteristics to active and sham tDCS groups will be assessed. Due to anticipated multicollinearity between PTSD symptoms and quality of life, we will adjust for baseline PTSD severity as the sole *a priori* covariate. Additionally, the degree to which Veterans report changes in other treatments during the intervention will be evaluated to ensure observed changes are related to tDCS+VR. Data analysis will calculate rates of dropout and distributional properties of dependent and other variables.

We will use generalized linear mixed models (GLMM) regressing follow-up (EOT) data on baseline and random treatment assignment in an ANCOVA model, while adjusting for baseline PTSD symptom severity (i.e., PCL score). This is a powerful approach to analyzing randomized controlled trials (Everitt & Wessely, 2008). The first step in analyses is selection of the proper link function and distribution family of the data driven by the scale of the response variable. Specifically, an identity link and Gaussian distribution family for normally distributed dependent variables, the Poisson distribution for count or proportion dependent variables, and log or logit link with binomial distribution for binary or ordered categorical dependent variables. However, when count data approaches normality, the Gaussian distribution may be appropriate. Conversely, when count data are overly dispersed (variance is greater than the mean), a negative binomial family may be required. To guide this decision making process, if the nature of dependent variable insufficiently indicates the appropriate link/family, we will inspect histograms, Q-Q plots and estimate Shapiro-Wilk tests of normality for dependent variables. However, we will not base decisions on P-values alone from the Shapiro-Wilk test, as this test while among the most powerful in assessing normality, is not fully efficient. Rather, we will use these devices as pieces of information to check modeling assumptions and inform alternative model selection. With a sense of the distribution, we will fit our GLMMs using two or more alternative families (e.g., Gaussian vs. Poisson vs. negative binomial) and contrast model fit using the deviance statistic. We will select the model with the smallest deviance statistic as the optimal model for the data. Also note we will estimate those models that seek to identify the optimal model with control-group data only, and will include only time as a predictor variable.

Related to our measurements, the PCL-5 is a 20-item questionnaire, with each item rated 0-4. It can be scored using item response theory methods (Bliese et al., 2008) in which case the derived scores are theoretically continuous normal. Bliese et al. (2008) also report in their samples of combat Veterans that raw sum scores are essentially normally distributed. Therefore we expect that a Gaussian family may be appropriate for the PCL-5, although a skewed distribution is possible and a Poisson or negative binomial model may be a better fit. Similarly, the total severity score of the CAPS-5, a 30-item diagnostic tool and a severity-rating instrument, may be normally distributed (Weathers et al., 1999), but deviations from that in our sample cannot be ruled out and will be investigated. Social and occupational function (SOF) will be assessed with the SOFAS, a clinician rated scale ranging from 1-100, and is also likely to be normally distributed in clinical samples. But, a log normal or negative binomial model may be needed given the truncated distribution of possible scores (Morosini et al., 2000). Quality of life (QOL) will be measured with the 18-item Quality of Life Enjoyment and Satisfaction Questionnaire (Endicott et al., 2005). The items are rated on a 5-point scale indicating frequency and are summed to form scores. The high number of items and response scale, and prior research in clinical samples also leads to an expectation that normal assumptions may be reasonable for this instrument (Ritsner et al., 2005). Finally, psychophysiology will include skin conductance reactivity (SCR) to specific trauma context VR cues presented in the VR scenario. SCR is typically not normally distributed and square root or log transformations will be applied following accepted SCR data approaches (Milad et al., 2005).

All analyses will use two-sided alpha = .05 and significance levels will be estimated using permutation test methods. Although not anticipated, if substantial attrition occurs we will perform sensitivity analyses with joint models including a longitudinal change component and survival (i.e., retention) component. In the inevitable event of missing data, we will use multiple imputations in accordance with recommendations of the National Research Council Panel on Handling Missing Data in Clinical Trials (2010). Sensitivity analyses will be used to examine the degree to which our inferences are subject to assumptions about mechanisms of missing data.

Aim 1: Prospectively evaluate the efficacy of tDCS+VR in Veterans with chronic PTSD.

Hypothesis Testing Aim 1: We will test the hypothesis that active tDCS+VR, compared to sham+VR results in greater change on primary outcomes, 1A) PCL and 1B) QLESQ. Testing hypothesis 1B focuses on end-of-treatment group (active tDCS vs. sham) differences controlling for baseline (PCL) differences. Our second objective is to test whether tDCS+VR results in sustained change in primary outcome measures at 1- and 3-months follow-up in the active tDCS group as compared to the sham group, again net of any baseline differences. We will use GLMM, with the follow-up observation of the outcome used as the dependent variable, and the baseline score, treatment assignment, stratification factors used in the urn randomization. We will include the follow-up time point as the dependent variable and include dummy variables for time point (omitting the dummy for EOT) and interactions with treatment assignment to identify differences between EOT treatment and the different time points. We will then repeat this analysis for clinician-evaluated measures of PTSD and quality of life (i.e., CAPS and SOFAS) following similar procedures. Effect of treatment status will be evaluated in an exploratory, follow-up analysis.

Aim 2: Evaluate the relationship between psychophysiology, PTSD symptoms and QOL/SOF.

Hypothesis Testing Aim 2: We will test whether (change in) psychophysiological responses relate to (changes in) PTSD symptoms and QOL/SOF measures differently after active tDCS+VR vs. sham+VR. Moreover, we will test whether early changes in psychophysiology (desensitization to VR trauma context) will predict PTSD and QOL/SOF improvements. Secondary outcomes will include evaluation of changes in other variables that are either hypothesized or directly affected by tDCS+VR (e.g., depression, sleep, and illness severity and improvement) over time. The analysis approach will be similar to that used in Aim 1, with a GLMM regression on psychophysiological responses, PTSD symptoms, QOL, and treatment assignment.

Exploratory Aim 3: Explore the relationship between neuroimaging, PTSD and QOL/SOF.

Hypothesis Testing Exploratory Aim 3: To explore the relationship between neuroimaging, PTSD, and QOL/SOF, we will first characterize whether active tDCS+VR is superior to sham tDCS+VR in increasing resting state functional connectivity between the VMPFC and amygdala. For imaging analyses, we will adjust for *a priori* covariates that include age, sex and baseline PTSD symptom severity. To evaluate the VMPFC to amygdala relationship, we will seed the basolateral amygdala (BLA), a key region involved in fear extinction learning (VanElzakker et al., 2014; Rosenkranz et al., 2003) with clear anatomical boundaries, identified using cytoarchitectural parcellation of Amunts et al. (2005) (conceptual model of VMPFC to amygdala connectivity shown at right). Average time-series data will be extracted from the BLA, and general linear modeling (GLM) will be used to quantify the relationship between the seeds and subsequent targets below. GLM results will yield individual R^2 values, which will be

normalized into Z scores using Fisher's R-to-Z transformation. The individual maps of Z scores will be the basic measure of connectivity between the seed and target regions of interest (ROIs). We will then implement a ROI to ROI analyses within CONN, to evaluate RSFC between the BLA and VMPFC. Regions of the VMPFC will be derived targets from standard, ICA-based decomposition based atlases inclusive of 499 functional brain regions (Richiardi et al., 2015); all analyses will be corrected for multiple comparisons using false-discovery rate (FDR) correction. To explore the relationship between this neural circuit and QOL/SOF, Pearson correlation coefficients will be generated to describe the relationship between Z scores of connectivity (i.e., change from pre- to post-treatment) with changes in PTSD symptoms and QOL/SOF, performed in SPSS (v21, Armonk, NY). Furthermore we will also explore whether imaging metrics at baseline predict future improvements in QOL/SOF associated with active tDCS+VR.

Exploratory Aim 4: Explore the individualization of transcranial direct current stimulation intensity based on individual brain anatomy to inform future research.

Hypothesis Testing Exploratory Aim 4: To explore whether variability in obtained electrical fields based on individual neuroanatomy can guide the implementation of a standardized tDCS dosage, we will use already-collected T1 structural MRI scans as part of this protocol. The MRIcro(n) tool will be used to convert the scans from DICOM format to NIFTI format. The open-source MATLAB tool "Realistic vOlumetric Approach to Simulate Transcranial electric stimulation" (ROAST) (Huang et al., 2019) will be used for electrical field modeling of the aforementioned T1 structural scan on an individual basis in order to obtain voltage gradients and electrical field magnitudes based upon prespecified tDCS parameters. ROAST also calls upon the open source packages GetDP and Iso2Mesh in MATLAB. Numerical values, i.e. voltage gradients and electrical field magnitudes, provided by ROAST will be stored in a compiled excel file on the server. tDCS montages for multiple brain regions will this way be simulated. No actual tDCS will be delivered as part of this aim, and this aim solely involves finite element modeling with results allowing to inform future studies.

EEG data (optional): Data will be analyzed in accordance with similar methods used by our group, inclusive of the use of EEG analysis software and programs (e.g. MATLAB, C++, Python, etc.). These may include scripts written by members of our research team. Data may be analyzed both at the electrode level and using source localization.

SOURCE OF MATERIALS

Research materials obtained from participants will be clinical diagnostic interviews, rating scales, and, when appropriate, a urine pregnancy test or drug screen. All data will be used solely for research purposes. All data collection will follow HIPAA guidelines. Data will be collected directly from subjects, subjects' charts, or their treatment providers by the investigator team that will include a doctorate-level interviewer and a bachelors-level research assistant. The research assistant will be trained in administering rating scales and Dr. Shea will supervise administration of and perform quality control for the standardized interviews. All study staff will have completed Providence VAMC-approved training in the responsible conduct of research, and the PI will ensure all training is completed and up to date. All data collected in this study will be treated as confidential, and will never be stored or reported in association with identifying information. Participant responses to interviews or rating scales will be collected and initially stored in a hard copy format, then entered and stored electronically.

RISKS/BENEFITS ASSESSMENT

Potential Risks

Risk of discomfort from questionnaires/interviews: There is a risk of discomfort when being asked to rate clinical symptoms. Participants may experience distress from being asked to recall memories of past trauma and answering questions about how much they are bothered by symptoms of PTSD and other difficulties in living. Mental health symptoms may temporarily worsen as a result of these questions, and some participants may feel as if their privacy has been invaded.

Risk of side effects from tDCS treatments: tDCS is considered a low risk technique that is well tolerated and carries minimal to no side effects; there is some inherent risk with the application of electrical current. Stimulation is accompanied by an itchy or prickly sensation on the skull under the electrodes. The most common side effect is some transient redness of the skin under the electrodes that dissipates quickly after tDCS offset. Skin irritations can occur and the most severe adverse event would include a local skin burn where the electrodes are attached.

tDCS is not recommended during pregnancy, therefore pregnant women are excluded from this study. The effect of tDCS on pregnant women and on a developing fetus is unknown and this treatment may be harmful to the developing fetus. Women of childbearing age will be inquired about pregnancy. When appropriate, the participant will be asked to undergo pregnancy testing prior to any study procedures. Confirmed pregnancy will lead to an immediate exclusion from the study.

A focus on seizure risk, tDCS and medications: Potential participants with medical/neurologic contraindications to stimulation are excluded, and it is required that all medications be stable for >6 weeks prior to participation. Furthermore, tDCS is thought to be safe without risk of seizure induction in the absence of neurologic risk factors. This is largely due to its nature as a low-intensity, sub-threshold stimulation. For example, the amount of current needed to induce a seizure (e.g., with electroconvulsive therapy) is approximately 800mA, whereas in this study we are using 2mA. tDCS has been shown to be safe even in patients with a seizure disorder (these participants are excluded from this study, described here only as an example of safety). Furthermore, a recent consensus statement, based on experience of over 33,000 tDCS sessions and over 1,000 subjects (inclusive of patient populations), stated that there is "no evidence of mechanistic support for seizure generation with conventional tDCS protocols (≤40 min stimulation, ≤4 mA stimulation intensity, ≤7.2 Coulombs)" as implemented in the current proposal (Bikson et al., 2016).

All medical aspects of tDCS administration will be supervised by the tDCS Attending Physician, (Noah S. Philip MD). Dr. Philip has received advanced training in neuromodulation, including tDCS, and directs the Psychiatric Neuromodulation Clinic at the Providence VAMC. tDCS procedures will be administered by the tDCS Operator, who is trained and qualified to deliver, or assist in delivery of tDCS. This individual is trained in the use of tDCS devices at the PVMAC or Butler Hospital, Providence RI. He/she has knowledge of safety considerations and precautions associated with tDCS.

Risk of side effects of virtual reality: Although exposure procedures are standard in the treatment of PTSD, exposure to potentially traumatic triggers (sights, sounds, smells) in the VR environment scenes are designed to produce short-term emotional reactions, and these

emotional reactions can be strong. In addition to discussing upsetting events or having emotional reactions, the participant may also experience symptoms of "cybersickness." Cybersickness is a term for types of side effect symptoms that may occur during participation in the virtual reality environment. Cybersickness includes three aspects: nausea, disorientation or postural instability, and visual symptoms (eyestrain and/or blurred vision). These symptoms can occur both during the VR procedure, as well as afterwards.

Risk of MRI: MRI imaging is generally considered to be safe, although bodily inclusion of metallic objects or exposure would present significant danger. There is a risk of claustrophobia during an MRI. There is a risk of heating from radiofrequency coils. There is a risk of distress from finding a previously unknown structural abnormality in the brain. There is a risk of hearing injury from the loud noise in MRI.

Risk of worsening symptoms or lack of improvement: Risks associated with participation in this trial include possible lack of positive response to tDCS+VR, or worsening of PTSD symptoms or fear response. There is no guarantee that the treatment will lead to improvement of symptoms. During the course of treatments or after finishing the final session, symptoms may worsen.

Risk of inconvenience and burden of required time/travel: Subjects may engage in screening procedures and learn they are not eligible for participation in the research treatment trial. Emotional discomfort may be associated with completing the assessments and questionnaires. Frequent visits to the research clinic for the tDCS+VR treatments (e.g. 3 days per week for two weeks) may represent an inconvenience, especially if a subject travels a great distance or has other constraints on their time or transportation. A small payment will be offered to cover part of the subject's expenses related to participation in this research study, but subjects will not be offered reimbursement for all of the expenses they may incur.

Confidentiality: There is some risk to patient confidentiality associated with participation in research clinical trials, as more data are collected than would happen in usual medical practice. Steps will be taken to protect privacy of patient health information. Patients will be told that information about their treatment may be shared with their prescribing mental health provider and/or primary care provider, per best-practice standards.

Rating scales: There is a risk of discomfort when being asked to rate clinical symptoms.

Risk of (optional) EEG: EEG is a non-invasive and safe examination in which no form of ionizing radiation or any injections, such as contrast agents, are needed. Electrical safety of EEG will be accounted for to reduce the risk of electrical shock. For electrical safety, most EEG systems operate off a battery pack that needs to be continually charged. Though no adverse effects of EEG have been reported, other than possible scalp irritation due to the placement of surface electrodes, it is possible that effects not yet reported may occur. The participant may not like having electrode gel in their hair.

Other: There may be other risks that are currently unknown. Although tDCS has been used for many years, the long-term effects of tDCS on individuals are not completely known. The research team will notify subjects if anything new is learned about the safety of tDCS that might make them change their mind about participating in this study.

Protection against Risk

Risk of discomfort from questionnaires/interviews: Participants will be encouraged to discuss any discomfort with the study procedures with one of the researchers. Patients are informed that they can refuse to answer any question or stop the assessment or treatment at any time. They will be informed that they can choose to resume the assessment or treatment or refuse to participate further. If a participant becomes distressed and requires additional support, a study staff member will put them in touch with their mental health care provider. The PI or their designate will be available at all times for assessments; both Drs. Philip and Greenberg and board-certified psychiatrists with experience treating Veterans with PTSD and the entire investigator team is familiar with emergency care procedures at the Providence VAMC. The participant will also be given a handout with the contact information for VA mental health emergency procedures, along with the PI's contact information, in the event the participant experiences any distress after leaving the session. Any treatment-emergent safety concerns, such as suicidality or homicidality, detected either on rating scales, or expressed to investigator team, will lead to an immediate on-site assessment by Drs. Philip or Greenberg, both of whom have experience dealing with PTSD and emergent psychiatric situations. If for any reason they are not immediately available Veterans will be escorted by VA police to emergency psychiatric facilities for further assessment (i.e., psychiatric Urgent Care run by Providence VAMC Mental Health, located on site).

Risk of side effects from tDCS treatments: Some tDCS adjustments may be possible to make the experience more comfortable. Over-the-counter pain medications such as acetaminophen or ibuprofen may be helpful to reducing discomfort from tDCS treatments. Assessment of the subjects' well-being and functioning before and after each treatment, special positioning and targeting procedures to ensure the tDCS is placed over the specific brain region in this study (prefrontal cortex), and constant monitoring during each treatment session, will all be done to minimize the risk of experiencing side effects.

To avoid skin burns, participants will be instructed to immediately signal any discomfort while receiving tDCS. Electrodes sponges attached to the scalp will be pre-moistened with saline or conductive gel and resistance will be checked throughout stimulation. The NeuroConn device uses a predetermined safety setting to not stimulate when a resistance of 55 k ohm is detected. User experience with this device has resulted that a resistance of 10 k ohm or less can easily be acquired. In safety testing of the electrodes, subjects were exposed to current densities of up to 2.56 mA/cm^2, and durations of up to 30 minutes. No burns or significant discomfort occurred. The use of saline or conductive gel prevents burns by distributing the current evenly over the surface of the electrode sponge. In addition, electrodes/sponges will not be placed over skin lesions, such as vascular moles and angiomas that might have greater conductance than the surrounding skin. DC polarization has been applied unilaterally to the primary motor and dorsal prefrontal areas in many studies over the past decade (Wassermann, 2008) with no reports of adverse effects attributable to effects on the central nervous system. The proposed stimulation intensity is similar to the stimulation intensity used in previous protocols as also performed at the NIH (and for which the risk determination from the FDA was requested). No adverse side effects have been reported in these previous studies with the proposed stimulation settings. The proposed stimulation intensity level and length is therefore at the level at which we will expect to find effects of tDCS on neural and behavioral output measures, without causing adverse side-effects. To date, we have performed tDCS on more than 100 participants, including Veterans

with a diagnosis of PTSD. No Veterans have been unable to tolerate tDCS procedures.

Risk of side effects from virtual reality: As indicated previously, careful monitoring and discussion with participants will mitigate the risks of clinical deterioration. During VR, participants will have a staff member in the procedure room, which will be monitoring vital signs. A psychiatrist with experience with PTSD will be available at all times. Participants will also be informed that they can stop VR procedures at any time, by saying the word “STOP.” With respect to “cybersickness”, the following procedures to minimize risk will be followed participants will be informed that these symptoms (lightheadedness, dizziness, upset stomach, or visual symptoms, like eyestrain and/or blurred vision) may occur in a small percentage of persons. Participants will be instructed to notify the research staff immediately during the VR procedures if any of these cybersickness symptoms are experienced. At that point, staff will stop the procedure and call the covering physician. In the unlikely event that symptoms persist, the participant will be taken to the Providence VA ER for evaluation. Providing the participant with the researchers' contact information, in addition to the follow-up call (3-7 days after the VR procedure) will also help to assess any problems the participant might experience after the procedure.

Risk of MRI: Subjects will be screened for metallic objects or exposure that would preclude them from participation in the study. Subjects will fill out standard questionnaires and informed consent for FMRI scanning. They will be informed that there is a risk of claustrophobia during the scan. Multiple measures will be used to ensure the safety and comfort of veterans during the scan sessions. MRI operators will monitor the participants using 2-way voice intercom, infrared camera, and monitoring psychophysiologic (i.e., pulse and respiration rate) data. Participants will additionally be able to utilize a squeeze bulb that will immediately indicate distress to the scanner operator. If needed, the PI is a Board-Certified Psychiatrist and can be available to assess clinical needs and make further decisions about referrals or more acute management needs. Subjects will be informed of the risk of heating from radiofrequency coils and instructed to inform the research technicians if this occurs. Subjects will also be informed that the scan session provided does not constitute a clinical scan and that researchers are not board- certified radiologists. If there is concern for a physical abnormality on structural scan, the PI will provide an appropriate referral. All serious treatment-emergent adverse events will be brought to the VA IRB as required in IRB protocols. The PI and key personnel will discuss all significant or potentially significant adverse events. Participants will be required to wear hearing protection during all MRI procedures.

Risk of Worsening Symptoms or lack of Improvement: The research staff will evaluate subjects at every treatment session, and a psychiatrist with experience with neuromodulation and PTSD (i.e., the PI or their representative) will be available at all times to ensure participation in this research continues to be safe and reasonable for them. This psychiatrist will be available during every session to assess any Veteran who has significant emotional distress, worsened fear response, or other difficulties with study procedures. After hours contact information, including emergency psychiatric access, will be provided to all participants. Principles of good clinical practices will be followed in every event of clinical deterioration.

Risk of Inconvenience and Burden of Required Time/Travel: A small payment will be offered to cover part of the subject’s expenses related to participation in this research study, but subjects will not be offered reimbursement for all of the expenses they may incur.

Confidentiality: Every effort to maintain participant confidentiality will be made. All research

personnel will be trained in the responsible conduct of research and the Principal Investigator will be responsible for ensuring that adequate training has been completed. Participants' records/assessments will not become a part of their permanent medical record. All study forms and data will be identified only by code numbers, and will be stored in locked file cabinets or on secure research servers and MRI data will be stored on scientific computing servers. Identifying information (contact information, name, consent documents) will be separated from the research data and be stored separately in a different locked file cabinet. All computerized data will be stored on secure, password-protected files. No personal participant information will be presented in any publication or presentations resulting from this research. Only the PI, investigator team and research assistants will have access to materials gathered during the study.

Telephone/Virtual Visits: Study staff will make every effort to protect the confidentiality of the participants during virtual study visits. Staff will use VA approved methods of communication such as VVC and telephone. Study staff will also instruct participants to find a private space for phone/video sessions so not to be overheard and to help keep participant information private.

Rating Scales: Subjects will be informed that if they experience discomfort during symptom assessment, study personnel will take appropriate measures, including debriefing and referral to the principal investigator for further evaluation.

The participants' well-being will always be placed above research considerations. Participants will understand that they can refuse to answer any question or to stop an interview at any time. The subjects will be clearly informed that they are free to terminate participation in the study at any point.

Risk of (optional) EEG: Participants will be informed that EEG is non-invasive with minimal risk of side effects and that the EEG portion of the study is optional and if they chose to participate, they can opt out at any time. Research staff will observe for potential scalp irritation after EEG electrodes are removed after the procedure, and ask participants to inform staff if irritation develops after leaving the research clinic, in which case staff will advise on local care for irritation or refer for treatment in the unlikely event that is needed.

<u>Benefits</u>

Benefits to subject participating in this trial include a thorough evaluation of PTSD, mood and anxiety symptoms at no cost, and possible relief from symptoms from tDCS+VR. The potential benefits of this project to others may include enhanced knowledge about treatment options for PTSD, about optimal tDCS use, and about outcomes among the type of patients often encountered in clinical practice but excluded from participation in previous large clinical trials of brain stimulation – those with chronic PTSD and comorbidities.

IMPORTANCE OF KNOWLEDGE TO BE GAINED

The results of this study will inform whether tDCS+VR can be an effective treatment to reduce symptoms of PTSD and improve quality of life. If successful, this research will lead to further development of tDCS plus other types of psychotherapy, resulting in a powerful and individualized rehabilitation option for Veterans with chronic PTSD. Therefore, in relation to the importance of the knowledge to be gained, the risk to participants is reasonable.

REPORTING OF SERIOUS OR UNEXPECTED ADVERSE EVENTS

For the purposes of this project, adverse events will be reported with the following information: Subject identification number, PI's name, age, gender, and ethnicity, dates of participation in the study, signs/symptoms and severity, date of onset, date of resolution or death, relationship to study intervention, action taken. Unanticipated problems involving risk to participants or other serious adverse events will be promptly reported to the Providence VA Medical Center's IRB. Reporting timeframes will be consistent with those established by the IRB.

MEDICAL CARE FOR RESEARCH RELATED INJURIES

The VA medical facility shall provide necessary medical treatment to research subjects injured as a result of participation in a research project. This project has been approved by a VA Research and Development Committee and will be conducted under the supervision of one or more VA employees in accordance with Federal regulations. Additional compensation may or may not be available under federal law. Further information regarding eligibility for medical care and compensation under federal law in the event of injury or illness may be obtained from the Medical Administration Service at (401) 273-7100 extension 3300. Participants may also contact the Medical Center's Patient Advocate at (401) 273-7100 extension 3093.

WITHDRAWAL/TERMINATION OF SUBJECTS

Early Termination: Subjects will be discontinued from the study if they: (a) require hospitalization for a psychiatric decompensation that precludes their attending the second session; (b) engage in an uncontrolled episode of alcohol or drug abuse that requires immediate treatment; (c) do not follow study procedures, which could be due to participants requesting termination or study team decision that study continuation might be unsafe. Any data collected prior to termination in the study will be included in data analyses. If the participant is terminated from the study by the PI, he will be thanked for his/her participation and the appropriate referrals will be made to ensure follow-up treatment/services are received, as needed.

Withdrawal: It will be made clear that participation in this research study is completely voluntary, and the participant may decide to stop his/her participation at any point in the study. A participant will be withdrawn from the entire study if, at any time during the study the PI feels that continuation in the study would be detrimental to the participant's condition, as evidenced by the participant needing hospitalization for increase of symptoms/his her decompensation of functioning or the participant so desires.

DATA SAFETY AND MONITORING PLAN

Weekly meetings with study staff and study investigators will be held to review progress with regard to enrollment, any adverse events, and attrition/noncompliance. Circumstances surrounding any identified adverse events, incidents of subject dissatisfaction, or subject noncompliance/withdrawal of consent will be tracked regularly and discussed. Adverse events tracking files will be routinely updated. If data patterns consistent with any safety issues are suggested, the principal investigator will seek consultation with, and peer review by, other experienced research colleagues who have executed similar studies with the methods and procedures of concern. Serious adverse events will be identified and promptly reported to the Providence VA IRB as required. A member of the research team will be on-site during all

sessions, and in the event of any subject becoming unstable or demonstrating clinical symptoms, the principal investigator and/or study team will assess the subject and facilitate subsequent treatment or referral. All research and staff members are trained in basic first aid, CPR, and appropriate brain stimulation safety/evacuation protocols. All members of the research team have 24-hour access to investigators or covering psychiatrists on site for management of any clinical emergency that may arise. To ensure the integrity of the data the PI and study team will review all the data for errors or inaccuracy within one week after it is obtained. All data will be entered into a research database as it is collected (i.e. RedCAP), and the research assistant will meet with PI weekly or as appropriate to review ongoing subject data. All records collected and stored will be maintained in accordance with Record Control Schedule 10-1.

ROLES AND RESPONSIBILITIES OF STUDY PERSONNEL

Research Assistant: The Research Assistant will be responsible for implementing recruitment procedures and managing study data. He/she will also coordinate study appointments and be responsible for scheduling participants, obtaining informed consent and HIPAA authorization, collecting screening data, and administering assessments.

tDCS Operator: a trained and qualified individual supervised by the tDCS Attending Physician (Dr. N. Philip) to deliver, or assist in delivery of, tDCS. This individual is trained in the use of tDCS devices at PVMAC or Butler Hospital, Providence RI. He/she has knowledge of safety considerations and precautions associated with tDCS. Responsibilities include:
A). Positioning of the tDCS device on the participant prior to initiation of stimulation.
B). Operation of the hardware associated with the tDCS device.
C). Administration of tDCS, at the parameters established by the tDCS Attending Physician.
D). Brief assessment of relevant mental status and general clinical condition before and after tDCS.
E) Monitoring of the participant during the tDCS session. A tDCS Operator must remain in the room and observe the participant's physical status for the potential occurrence of adverse events throughout the entire tDCS session.
F). Making routine adjustments to the placement of the device as required and consistent with product labeling (e.g., to ensure contact between participant's head and electrode) during the tDCS session. The tDCS Operator may not independently make any revision to pre-determined stimulation dose or electrode position parameters prescribed by the tDCS Attending Physician.
G). Determination of circumstances under which tDCS should be interrupted or terminated (e.g., participant expresses increasing discomfort under the electrodes; observation of participants for signs of skin burns, discomfort or other stress; participant wants to discontinue study procedures).
H). Administration and collection of self-report assessment forms.
I). Taking action in accordance with established regulations in case of adverse events, e.g. report of adverse event (skin lesion or significant skin discomfort) to the tDCS Attending Physician, or PI (if tDCS Operator is different from PI); seek medical attention if necessary; if there is any doubt about the mental or physical status of an individual after testing, the tDCS Attending Physician will evaluate the participant and make a recommendation for follow-up care if that is required. Telephone or in-person follow-ups will be arranged as needed. Any participant judged on clinical grounds to have suffered adverse effects will thus be evaluated and treated as necessary and withdrawn from the study.

REFERENCES

Amunts K, Kedo O, Kindler M, Pieperhoff P, Mohlberg H, Shah NJ, … Zilles K (2005). Cytoarchitectonic mapping of the human amygdala, hippocampal region and entorhinal cortex: intersubject variability and probability maps. Anatomy and Embryology 210: 343–352.

Asthana M, Nueckel K, Mühlberger A, Neueder D, Polak T, Domschke K, … Herrmann MJ (2013). Effects of transcranial direct current stimulation on consolidation of fear memory. Frontiers in Psychiatry 4: 107.

Baek K, Chae JH, Jeong J (2012). The effect of repetitive transcranial magnetic stimulation on fear extinction in rats. Neuroscience 200: 159–165.

Bikson M, Grossman P, Thomas C, Zannou AL… Woods AJ (2016). Safety of Transcranial Direct Current Stimulation: Evidence Based Update 2016. Brain Stimulation 9:641-61.

Blatt SJ, Zuroff DC, Bondi CM, Sanislow CA, Pilkonis, PA (1998). When and how perfectionism impedes the brief treatment of depression: further analyses of the National Institute of Mental Health Treatment of Depression Collaborative Research Program. Journal of consulting and clinical psychology 66: 423.

Blake, D., Weathers, F. W., Nagy, L. M., Kaloupek, D. G., Gusman, F. D., Charney, D. S., & Keane, T. M. (1995). The development of a clinician-administered PTSD scale. *Journal of Traumatic Stress, 8*, 75-90.

Bliese PD, Wright KM, Adler AB, Cabrera O, Castro CA, Hoge CW (2008). Validating the primary care posttraumatic stress disorder screen and the posttraumatic stress disorder checklist with soldiers returning from combat. *Journal of Consulting and Clinical Psychology* 76: 272–281.
Bremner JD et al (2005). Positron emission tomographic imaging of neural correlates of a fear acquisition and extinction paradigm in women with childhood sexual-abuse-related post-traumatic stress disorder. *Psychological medicine* 35: 791-806.

Buysse DJ, Reynolds CF, Monk TH, Berman SR, Kupfer DJ (1989). The Pittsburgh Sleep Quality Index (PSQI): A new instrument for psychiatric research and practice. *Psychiatry Research* 28: 193-213.

Carlson, E. B., & Putnam, F. W. (1993). Dissociation: Vol. 6, No. 1, p. 016-027: An update on the Dissociative Experience Scale.

Coffman BA, Clark VP, Parasuraman R (2014). Battery powered thought: enhancement of attention, learning, and memory in healthy adults using transcranial direct current stimulation. *Neuroimage* 85: 895-908.

Difede J, Hoffman H, Jaysinghe N (2002). Innovative use of virtual reality technology in the treatment of PTSD in the aftermath of September 11. *Psychiatric Services* (Washington, D.C.) 53: 1083–1085.

Etkin A, Wager TD (2007). Functional neuroimaging of anxiety: a meta-analysis of emotional processing in PTSD, social anxiety disorder, and specific phobia. *Am J Psychiatry* 164: 1476-88.

Everitt BS, Wessely S. (2008) *Clinical trials in psychiatry*. John Wiley & Sons. Finn ES, Shen X, Scheinost D, Rosenberg MD, Huang J, Chun MM, ... Constable RT (2015). Functional connectome fingerprinting: identifying individuals using patterns of brain connectivity. *Nature Neuroscience* 18: 1664–1671.

First MB, Spitzer RL, Gibbon M, Williams JBW (1994). Structural Clinical Interview for Axis I DSM-IV Disorders: Biometrics Research Department. New York State Psychiatric Institute, New York.

Foa EB, Huppert JD, Cahill SP (2006). Emotional processing theory: An update. In Rothbaum BO (Ed.), *Pathological anxiety: Emotional processing in etiology and treatment* (pp. 3–24). New York, NY: Guilford.

Foa EB, Kozak MJ (1986). Emotional processing of fear: exposure to corrective information. *Psychological bulletin* 99: 20-35.

Foa EB, Keane TM, Friedman MJ, Cohen JA (2008). Effective treatments for PTSD: practice guidelines from the International Society for Traumatic Stress Studies. Guilford Press. Forbes D, Creamer M, Biddle D (2001). The validity of the PTSD checklist as a measure of symptomatic change in combat-related PTSD. *Behav Res Ther* 39:977-86.

Guy W, editor. ECDEU Assessment Manual for Psychopharmacology. (1976). Rockville, MD: US Department of Heath, Education, and Welfare Public Health Service Alcohol, Drug Abuse, and Mental Health Administration.

Hoge CW, Castro CA, Messer SC, McGurk D, Cotting DI, Koffman RL (2004). Combat duty in Iraq and Afghanistan, mental health problems, and barriers to care. *The New England Journal of Medicine* 351: 13–22.

Huang et al. (2019). Realistic volumetric-approach to simulate transcranial electric stimulation—ROAST—a fully automated open-source pipeline. Journal of neural engineering, 16, 056006.

Ironside M, O'Shea J, Cowen PJ, Harmer CJ (2016). Frontal Cortex Stimulation Reduces Vigilance to Threat: Implications for the Treatment of Depression and Anxiety. *Biological Psychiatry* 79: 823–830.

Karsen EF, Watts BV, Holtzheimer PE (2014). Review of the effectiveness of transcranial magnetic stimulation for post-traumatic stress disorder. *Brain Stimul* 7: 151-7.

Koch SBJ, van Zuiden M, Nawijn L, Frijling JL, Veltman DJ, Olff, M (2016). Aberrant resting-state brain activity in posttraumatic stress disorder: a meta-analysis and systematic review. *Depression and Anxiety* Jul;33(7):592-605.

Marin MF, Lonak SF, Milad MR (2015). Augmentation of Evidence-Based Psychotherapy for PTSD With Cognitive Enhancers. *Current Psychiatry Reports* 17: 39.

Milad MR, Quirk GJ (2002). Neurons in medial prefrontal cortex signal memory for fear extinction. *Nature* 420: 70-74.

Milad MI, Quirk G (2004). Electrical stimulation of medial prefrontal cortex reduces conditioned fear in a temporally specific manner. *Behavioral neuroscience* 118: 389.

Milad MR, Vidal-Gonzalez I, Quirk GJ (2004). Electrical stimulation of medial prefrontal cortex reduces conditioned fear in a temporally specific manner. *Behavioral neuroscience* 118: 389.

Milad MR, Quinn BT, Pitman RK, Orr SP, Fischl B, Rauch SL (2005). Thickness of ventromedial prefrontal cortex in humans is correlated with extinction memory. *Proc Natl Acad Sci USA* 102: 10706–11.

Milad MR, Wright CI, Orr SP, Pitman RK, Quirk GJ, Rauch SL (2007). Recall of fear extinction in humans activates the ventromedial prefrontal cortex and hippocampus in concert. *Biol Psychiatry* 62: 446–54.

Milad MR, Orr SP, Lasko NB, Chang Y, Rauch SL, Pitman RK (2008). Presence and acquired origin of reduced recall for fear extinction in PTSD: results of a twin study. *Journal of psychiatric research* 42: 515-520.

Milad MR, Pitman RK, Ellis CB, Gold AB, Shin LM, Lasko NB, Zeidan MA, Handwerger K, Orr SP, Rauch SL (2009). Neurobiological basis of failure to recall extinction memory in posttraumatic stress disorder. *Biological Psychiatry* 15: 1075-1082.

Milad MR. et al (2009). Neurobiological basis of failure to recall extinction memory in posttraumatic stress disorder. *Biological psychiatry* 66: 1075-1082.

Milad MR Quirk GJ (2012). Fear extinction as a model for translational neuroscience: ten years of progress. *Annual review of psychology*63:129-151.

Moliadze V, Antal A, Paulus W (2010). Electrode-distance dependent after-effects of transcranial direct and random noise stimulation with extracephalic reference electrodes. Clinical Neurophysiology: Official Journal of the International Federation of Clinical Neurophysiology 121: 2165–2171.

Morosini PL, Magliano L, Brambilla L, Ugolini S, Pioli R (2000). Development, reliability and acceptability of a new version of the DSM-IV Social and Occupational Functioning Assessment Scale (SOFAS) to assess routine social functioning. Acta Psychiatrica Scandinavica 101: 323–329.

Mungee A, Kazzer P, Feeser M, Nitsche MA, Schiller D, Bajbouj M (2014). Transcranial direct current stimulation of the prefrontal cortex: a means to modulate fear memories. Neuroreport 25: 480–484.

Nasseri P, Nitsche MA, Ekhtiari H (2015). A framework for categorizing electrode montages in transcranial direct current stimulation. Frontiers in human neuroscience 9.

Nitsche, M. A., Cohen, L. G., Wassermann, E. M., Priori, A., Lang, N., Antal, A., Paulus, W., Hummel, F., Boggio, P.S., Frengi, F. & Pascual-Leone, A. (2008). Transcranial direct current stimulation: State of the art 2008. Brain stimulation, 1, 206-223.

Opris D, Pintea S, García S, Palacios A, Botella C, Szamosközi, & David D (2012). Virtual reality exposure therapy in anxiety disorders: a quantitative meta-analysis. *Depression and anxiety*, 29: 85-93.

Osuch, E. A., Benson, B. E., Luckenbaugh, D. A., Geraci, M., Post, R. M., & McCann, U. (2009). Repetitive TMS combined with exposure therapy for PTSD: a preliminary study. Journal of anxiety disorders, 23, 54-59.

Phelps EA, Delgado MR, Nearing KI, LeDoux JE (2004). Extinction learning in humans: role of the amygdala and vmPFC. Neuron 43: 897-905.

Philip NS, Ridout SJ, …, Carpenter LL (2016a). 5-Hz Transcranial Magnetic Stimulation for Comorbid Posttraumatic Stress Disorder and Major Depression. Journal of Traumatic Stress 29: 93–96.

Philip NS, Barredo J, , Tyrka AR, Price LH, Carpenter LL (2016b). Repetitive Transcranial Magnetic Stimulation Corrects Network Pathology in Patients with Comorbid Posttraumatic Stress Disorder and Major Depressive Disorder. Annual Meeting of the Society for Biological Psychiatry, Atlanta GA; in review at Biological Psychiatry.

Philip NS, Nelson B, Frohlich F, Lim K, Widge A, and Carpenter LL (2017). Low-Intensity Transcranial Current Stimulation in Psychiatry. American Journal of Psychiatry doi: 10.1176/appi.ajp.2017.16090996. Epub ahead of print

Power JD, Barnes KA, Snyder AZ, Schlaggar BL, Petersen SE (2012). Steps toward optimizing motion artifact removal in functional connectivity MRI; a reply to Carp. Neuroimage 76: 439-41.

Quirk GJ, Garcia R, González-Lima F (2006). Prefrontal Mechanisms in Extinction of Conditioned Fear. *Biological Psychiatry* 60: 337-343.

Rauch, S. L., Shin, L. M., Segal, E., Pitman, R. K., Carson, M. A., McMullin, K., Whalen P. J.,&Makris, N. (2003). Selectively reduced regional cortical volumes in post-traumatic stressdisorder. Neuroreport, 14, 913-916.

Richiardi J, Altmann A, Milazzo AC, Chang C, Chakravarty MM, Banaschewski T (2015). Correlated gene expression supports synchronous activity in brain networks. Science 348: 1241–1244.

Ritsner M, Kurs R, Gibel A, Ratner Y, Endicott J (2005). Validity of an abbreviated quality of life enjoyment and satisfaction questionnaire (Q-LES-Q-18) for schizophrenia, schizoaffective, and mood disorder patients. Quality of Life Research: An International Journal of Quality of Life Aspects of Treatment 14: 1693–1703.

Rosenkranz JA, Moore H, Grace AA (2003). The Prefrontal Cortex Regulates Lateral Amygdala Neuronal Plasticity and Responses to Previously Conditioned Stimuli. The Journal of Neuroscience 23: 11054- 11064.

Rothbaum BO, Davis M (2003). Applying learning principles to the treatment of Post-Trauma reactions. Annals of the New York Academy of Sciences 1008: 112-121.

Rothbaum BO, Price M, Jovanovic T, ..., Ressler KJ (2014). A randomized, double-blind evaluation of D- cycloserine or alprazolam combined with virtual reality exposure therapy for posttraumatic stress disorder in Iraq and Afghanistan War veterans. American Journal of Psychiatry 171: 640-648.

Rougemont-Bücking A, et al (2011). Altered processing of contextual information during fear extinction in PTSD: an fMRI study. CNS neuroscience & therapeutics 17: 227-236.

Rush AJ, Trivedi MH, Ibrahim HM, ..., Keller MB (2003). The 16-Item Quick Inventory of Depressive Symptomatology (QIDS), clinician rating (QIDS-C), and self-report (QIDS-SR): a psychometric evaluation in patients with chronic major depression. Biol Psychiatry 54: 573-83.

Russo R, Wallace D, Fitzgerald PB, Cooper NR (2013). Perception of comfort during active and sham transcranial direct current stimulation: a double blind study. Brain Stimulation 6: 946–951.

Sheehan, D.V., Lecrubier, Y., Sheehan, K.H., Amorim, P., Janavs, J., Weiller, E., Hergueta, T., Baker, R., Dunbar, G.C. (1998). The Mini-International Neuropsychiatric Interview (M.I.N.I.): the development and validation of a structured diagnostic psychiatric interview for DSM-IV and ICD-10. Journal of Clinical Psychiatry, 59, Suppl 20:22-33.

Shin, L. M., Wright, C. I., Cannistraro, P. A., Wedig, M. M., McMullin, K., Martis, B., Macklin, M. L., Lasko, N. B., Cavanagh, S. R., Krangel, T. S., Orr, S. P., Pitman, R. K., Whalen, P. J., & Rauch, S. L. (2005) A functional magnetic resonance imaging study of amygdala and medial prefrontal cortex responses to overtly presented fearful faces in posttraumatic stress disorder. Archives of General Psychiatry, 62, 273–281.

Spielberg CD, Gorsuch RL, Lushene RE (1983). Manual for the State-Trait Anxiety Inventory Consulting. Consulting Psychologists Press, Palo Alto, California.

VanElzakker MB, et al (2014). From Pavlov to PTSD: the extinction of conditioned fear in rodents, humans, and anxiety disorders. Neurobiology of learning and memory 113: 3-18.

van 't Wout M, Mariano TY, Garnaat SL, Reddy MK, Rasmussen SA, Greenberg BD (2016). Can Transcranial Direct Current Stimulation Augment Extinction of Conditioned Fear? Brain Stimulation 9: 529–536.

van 't Wout M, Longo SM, Reddy MK, Philip NS, Bowker MT, Greenberg BD. Transcranial direct current stimulation may modulate extinction memory in Veterans with posttraumatic stress disorder. Brain and Behavior 2017 (in press, doi: 10.1002/brb3.681)

Wassermann EM (2008) Direct current brain polarization. In: The Oxford Handbook of Transcranial Stimulation (Wassermann EM, Epstein CM, Lisanaby SH, Paus T, Walsh V, Ziemann U, eds), pp 57-61. Oxford: Oxford University Press.

Watson, D.,Clark, L. A., & Tellegen, A. (1988). Development and validation of brief measures of positive and negative affect: The PANAS scales. Journal of Personality and Social Psychology, 54(6), 1063-1070.

Weathers FW, Blake DD, Schnurr PP, Kaloupek DG, Marx BP, Keane TM (2013a). The Clinician-Administered PTSD Scale for DSM-5 (CAPS-5).

Weathers FW, Blake DD, Schnurr PP, Kaloupek DG, Marx BP, Keane TM (2013b). The PTSD Checklist for DSM-5 (PCL-5). Instrument available from the National Center for PTSD.

Whitfield-Gabrieli S, Nieto-Castanon A (2012). Conn: a functional connectivity toolbox for correlated and anticorrelated brain networks. Brain Connectivity 2: 125–141.

Wiederhold BK, Rizzo AS (2005). Virtual reality and applied psychophysiology. Appl Psychophysiol Biofeedback 30: 183-185.
