# Supplementary material for: Virtual Reality and Transcranial Direct Current Stimulation for Posttraumatic Stress Disorder: A Randomized Clinical Trial
Source: JAMA Psychiatry. 2024 Mar 6;81(5):437–46. doi: 10.1001/jamapsychiatry.2023.5661 (PMC10918574; doi:10.1001/jamapsychiatry.2023.5661)
Supplement: Supplement 2. — eTable 1. Service-Connected Conditions eTable 2. Trauma Types eTable 3. Side Effects by tDCS Group eFigure. Methodological Overview eAppendix 1. Staff Blinding eAppendix 2. Randomization eAppendix 3. Cluster Analysis for Clinical Outcomes eAppendix 4. Psychophysiology eAppendix 5. Impact of COVID-19 Pandemic [file jamapsychiatry-e235661-s002.pdf]

Supplemental Online Content

van ’t Wout-Frank M, Arulpragasam AR, Faucher C, et al. Virtual reality and transcranial direct current stimulation for posttraumatic stress disorder: a randomized clinical trial. *JAMA Psychiatry*. Published online March 6, 2024. doi:10.1001/jamapsychiatry.2023.5661

eTable 1. Service-Connected Conditions
eTable 2. Trauma Types
eTable 3. Side Effects by tDCS Group
eFigure. Methodological Overview
eAppendix 1. Staff Blinding
eAppendix 2. Randomization
eAppendix 3. Cluster Analysis for Clinical Outcomes
eAppendix 4. Psychophysiology
eAppendix 5. Impact of COVID-19 Pandemic

This supplemental material has been provided by the authors to give readers additional information about their work.

© 2024 van ’t Wout-Frank M et al. *JAMA Network Open.*

A. Supplemental Table 1. Service-Connected Conditions [a]

	Active (n=26)		Sham (n=28)	
	N	%	N	%
Approval Status				
Approved	24	92	25	89
Applied, Pending	<3	-	<3	-
Applied, Denied	0	0	<3	-
Never Applied	<3	-	<3	-
Approved %				
0-10%	0	0	0	0
11-20%	0	0	0	0
21-30%	0	0	0	0
31-40%	0	0.0	<3	-
41-50%	<3	-	3	12
51-60%	<3	-	<3	-
61-70%	<3	-	<3	-
71-80%	8	33	3	12
81-90%	3	13	7	28
91-100%	9	38	7	28
Reported Service-Connected Conditions				
Mental Health	22	92	23	92
PTSD	20	83	13	60
Depression	8	33	5	20
Anxiety	4	17	<3	-
All other or unspecified	<3	-	9	36
Physical Health	14	54.2	14	52.0
Tinnitus/ Hearing	7	29.2	5	24.0
Migraines	<3	-	<3	-
TBI	3	12.5	<3	-
Chronic Pain	3	12.5	4	16.0
All other conditions			6	
Condition(s) not indicated	0		<3	-

[a] Some total(s) are not reported because there were too few patients to provide numbers without compromising identifiability

© 2024 van 't Wout-Frank M et al. *JAMA Network Open.*

B. Supplemental Table 2. Trauma Types

	Active (n=26)	Sham (n=28)
Life Events Checklist (LEC) Items, *n* (%)[a,b]		
Natural disaster	22 (85)	25 (89)
Fire/Explosion	23 (88)	25 (89)
Transportation accident	25 (96)	26 (93)
Serious accident at work, home, or during recreational activity	21 (81)	23 (82)
Exposure to toxic substance	17 (65)	15 (54)
Physical attack	22 (85)	23 (82)
Assault with a weapon	22 (85)	23 (82)
Sexual assault	6 (23)	11 (39)
Other unwanted/uncomfortable sexual experience	6 (23)	8 (29)
Combat/war-zone exposure	23 (88)	25 (89)
Captivity	<3 (-)	6 (21)
Life-threatening illness/injury	16 (62)	20 (71)
Severe human suffering	19 (73)	20 (71)
Sudden violent death	17 (65)	18 (64)
Sudden accidental death	15 (58)	18 (64)
Serious injury, harm, or death you caused to someone else	14 (54)	14 (50)
Other very stressful event/experience(s)	15 (58)	18 (64)

[a] LEC items endorsed by subjects as "Happened to me" and/or "Witnessed" and/or "Part of my Job."
Totals equal greater than 100% due to multiple responses.
[b] Some total(s) are not reported because there were too few patients to provide numbers without compromising identifiability

© 2024 van 't Wout-Frank M et al. *JAMA Network Open.*

C. Supplemental Table 3. Side effects by tDCS Group

	Active (n=26)	Sham (n=28)
**Side Effect** [a]		
Headache	5 (19.2%)	11 (39.3%)
Neck Pain	5 (19.2%)	6 (21.4%)
Head Pain	<3 (-)	3 (10.7%)
Scalp Pain	5 (19.2%)	<3 (-)
Tingling	18 (69.2%)	16 (57.1%)
Itching	17 (65.4%)	7 (25.0%)
Burning	13 (50.0%)	8 (28.5%)
Redness	15 (57.7%)	10 (35.7%)
Ringing or Buzzing in Ears	4 (15.4%)	8 (28.5%)
Change in Mood	11 (42.3%)	18 (64.3%)
Changes in Concentration	8 (30.8%)	12 (42.9%)
Sleepy, Drowsy or Fatigue	9 (34.6%)	13 (46.4%)
Flickering Lights	<3 (-)	0 (0.0%)
Blurry Vision	4 (15.4%)	7 (25.0%)
Dizziness or Lightheadedness	<3 (-)	8 (28.6%)
Nausea	4 (15.4%)	10 (35.7%)

No significant group differences were observed (all ps>.1).
[a] Some total(s) are not reported because there were too few patients to provide numbers without compromising identifiability

© 2024 van 't Wout-Frank M et al. *JAMA Network Open.*

D. Supplemental Figure 1. Methodological overview

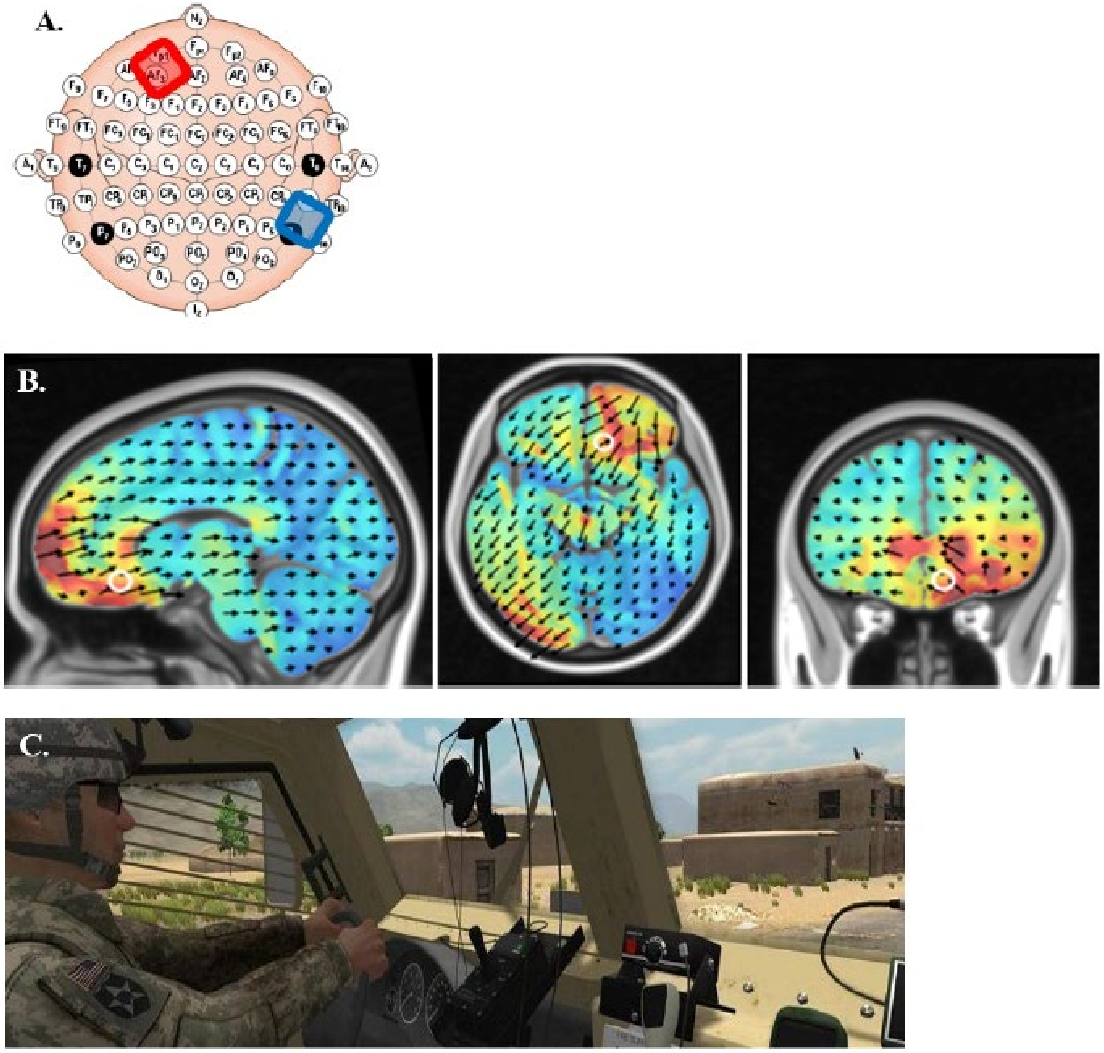

Figure Legend. Using a parallel-group double-blind, sham controlled design, participants received combined active transcranial direct current stimulation (tDCS) or sham stimulation + virtual reality (VR) for up to six sessions over 10 business days. tDCS was 2mA delivered using two 3x3cm^2 sponges at with the anode over 10-20 EEG coordinate AF7/Fp1/AF3 and the cathode between EEG coordinate OZ and the contralateral mastoid (covering approximately PO8/P8)(**A**). This resulted in a current density of ~2.22 A/m^2 in the ventromedial prefrontal cortex (image generated using Soterix HD-Explore Neurotargeting software) (**B**). In this example image, warmer colors indicate higher A/m^2 and cooler colors represent lower energy. VR was delivered using the Bravemind VR application (v. 1.0.4 or higher) from Virtually Better (Decatur, GA) developed by Albert “Skip” Rizzo PhD (USC/ICT). Screenshot of an example driving scenario used with permission (**C**). For further methodological details, see van ‘t Wout-Frank and Philip (2021).

© 2024 van ’t Wout-Frank M et al. *JAMA Network Open.*

1. Supplement: Staff Blinding

In prior blinded non-invasive brain stimulation studies for PTSD, we found that asking the administering staff to guess treatment assignment can be counterproductive. Because staff know they will be asked about group assignments at the end, they may look for elements that would inform their decision one way or another. This becomes additionally problematic as participants ask staff for their opinion, and if the staff is also trying to figure it out, this inevitable process invalidates the participant's guess at the end. It has been our experience from other tDCS protocols that asking staff to guess condition increases their chances to unblind, e.g., staff would start to look for cues such as skin redness which tends to be greater after active tDCS. Based on this experience, we do not set up queries for our staff at the end of participation and have careful instructions that staff never talk about group assignment.

2. Supplement: Randomization

The study protocol indicates use of mTBI and depression and stratification variables. These were erroneous retained. Prior to randomization we removed TBI and depression as stratification variables. This was because they are highly collinear with each other and PTSD in our local Veteran sample (e.g., see Philip et al., AJP 2019) and – had we implemented it – would have yielded 16 separate randomization subgroups.

3. Supplemental results: Cluster analysis for clinical outcomes.

A cluster consisting of 21 participants reported an average 24.3 (SD 6.79) point reduction on the PCL-5 indicating large and significant improvements compared to a second cluster of 33 participants reporting an average 1.32 (SD 7.50)-point reduction indicating no change (t=11.35, df=52, p<.001). The cluster reporting large improvement was predominantly participants allocated to active tDCS (14/21; 66.7%) versus sham (7/21; 30%), whereas significantly more participants allocated to sham (21/33; 63.6%) versus active tDCS (12/33; 36.6%) fell in the cluster reporting no change (Chi-Square=7.20, df=1, p=.03). On the CAPS, PTSD severity did not differ between groups at either 1- or 3-month follow-up, although there was an large effect size favoring active tDCS+VR at 3 months (t=-1.76, p=.08, Cohen's d =-.91

4. Supplemental results: Psychophysiology

We performed a sensitivity analysis including all participants, regardless of the quality of their skin conductance data. Using this approach, reductions in skin conductance reactivity across sessions was greater for the active tDCS + VR group compared to the sham + VR group, evidenced by a significant session by group interaction (F(5,10048)=2.53, p=.03). This demonstrates the augmentation by active tDCS on between-session habituation over VR sessions and robustness of the psychophysiology finding to questions regarding data quality. Furthermore, significant main effects further indicated that participants habituation across six sessions as well as within sessions independent of group (F(5,10048)=33.31, p<.001; F(2,10038)=6.38, p=.002, respectively), reflecting general efficacy of VR-based exposure. A significant main effect of discrete VR events demonstrated that different VR events elicited different responses as predicted (F(11,10038)=240.74, p<.001). However, the interactions between group and runs within session or discrete VR events were nonsignificant (both ps>.37), demonstrating that tDCS did not augment within-session habituation and groups did not differ in reactivity to specific VR events across all participants. There was also no clear effect of medications on SCR outcomes (all exploratory ps<.1).

© 2024 van 't Wout-Frank M et al. *JAMA Network Open.*

Our SCR analysis utilizes Trough To Peak (TTP) scoring in response to discreet VR events as well as assessing a 2-minute ‘baseline’ to capture tonic skin conductance level (SCL) prior to the start of each VR session. This makes testing for changes in tonic and phasic SCL across and within sessions challenging and we hesitate to make definitive conclusions. Mainly, after the end of each VR session, we immediately checked in with the participant and checked the tDCS device to ensure adequate conductivity was maintained throughout each session and did not assess tonic SCL post VR. Therefore, we were not able assess change in tonic SCL after each VR session. However, to provide the reviewer with additional insight into our data, we found no significant tDCS Group x VR session baseline SCL interaction (p=.63) and no significant main effects of tDCS Group (p=.34) or VR session baseline SCL (p=.57), suggesting no differences in baseline SCL between groups and across the six VR sessions.

5. Supplement: Impact of COVID Pandemic

This study was significantly impacted by the COVID pandemic. The study was written and funded prior to COVID, was stopped for significant periods due to the initial wave of COVID, and impacted by subsequent waves of infection. One way this clearly impacted the study was recruitment; the original target goal was 90 participants, but this became impossible after the shut down and resumption of research with significant safety procedures. We have explored whether outcomes are different in the pre- and “post”-COVID times, and while there were no clear findings, it is important to recognize COVID had a substantial impact on the study, including recruitment and likely related to quality-of-life outcome measures.

Furthermore, even with the reduced sample size the study had adequate statistical power. The original study was powered to detect an effect size of Cohen’s d= .53 for PCL-5 outcomes at the 1-month timepoint, assuming two groups of n=45. Using the same calculation with the reduced sample size yielded adequate power to detect an effect size of at least Cohen’s d = .73.

© 2024 van ’t Wout-Frank M et al. *JAMA Network Open.*
