# Supplementary material for: Virtual Reality and Transcranial Direct Current Stimulation for Posttraumatic Stress Disorder: A Randomized Clinical Trial
Source: JAMA Psychiatry. 2024 Mar 6;81(5):437–46. doi: 10.1001/jamapsychiatry.2023.5661 (PMC10918574; doi:10.1001/jamapsychiatry.2023.5661)
Supplement: Supplement 3. — Data Sharing Statement [file jamapsychiatry-e235661-s003.pdf]

Data Sharing Statement

van 't Wout-Frank. Virtual Reality and Transcranial Direct Current Stimulation for Posttraumatic Stress Disorder. *JAMA Psychiatry*. Published March 06, 2024.
doi:10.1001/jamapsychiatry.2023.5661

Data
Data available: No

Additional Information
Explanation for why data not available: Because this was a VA-funded study, data is available upon request to the authors subject to US Dept of Veterans Affairs rules & regulations.
